# Diversity and Biogeography of Bathyal and Abyssal Seafloor Bacteria

**DOI:** 10.1371/journal.pone.0148016

**Published:** 2016-01-27

**Authors:** Christina Bienhold, Lucie Zinger, Antje Boetius, Alban Ramette

**Affiliations:** HGF-MPG Group for Deep Sea Ecology and Technology, Alfred Wegener Institute Helmholtz Center for Polar and Marine Research, Bremerhaven, Germany, and Max Planck Institute for Marine Microbiology, Bremen, Germany; U.S. Geological Survey, UNITED STATES

## Abstract

The deep ocean floor covers more than 60% of the Earth’s surface, and hosts diverse bacterial communities with important functions in carbon and nutrient cycles. The identification of key bacterial members remains a challenge and their patterns of distribution in seafloor sediment yet remain poorly described. Previous studies were either regionally restricted or included few deep-sea sediments, and did not specifically test biogeographic patterns across the vast oligotrophic bathyal and abyssal seafloor. Here we define the composition of this deep seafloor microbiome by describing those bacterial operational taxonomic units (OTU) that are specifically associated with deep-sea surface sediments at water depths ranging from 1000–5300 m. We show that the microbiome of the surface seafloor is distinct from the subsurface seafloor. The cosmopolitan bacterial OTU were affiliated with the clades JTB255 (class *Gammaproteobacteria*, order *Xanthomonadales*) and OM1 (*Actinobacteria*, order *Acidimicrobiales*), comprising 21% and 7% of their respective clades, and about 1% of all sequences in the study. Overall, few sequence-abundant bacterial types were globally dispersed and displayed positive range-abundance relationships. Most bacterial populations were rare and exhibited a high degree of endemism, explaining the substantial differences in community composition observed over large spatial scales. Despite the relative physicochemical uniformity of deep-sea sediments, we identified indicators of productivity regimes, especially sediment organic matter content, as factors significantly associated with changes in bacterial community structure across the globe.

## Introduction

The deep ocean floor comprising the lower continental margin and abyssal plains at >1000 m water depth covers about half of Earth’s surface. Deep-sea surface sediments of the top 2 cm consist mostly of clay minerals, shells of planktonic organisms and organic matter; the benthic communities inhabiting the deep-sea floor are dominated by bacteria in terms of total organism abundance and biomass [1, 2], as well as in carbon and nutrient recycling and oxygen fluxes [3, 4]. Hence, the characterization of the composition and structure of bacterial communities, as well as their patterns of distribution, can provide important insights into the ecological and biogeochemical functioning of this vast ecosystem [5, 6]. In particular, the identification of relevant bacterial groups and of their functions in deep-sea sediments are key to understanding matter fluxes in deep-sea ecosystems and feedback mechanisms to environmental change and impacts [5, 7–9].

In recent years, the concept of microbiomes has emerged, whereby the collective entities of microorganisms and their genes typical for a specific host, habitat or ecosystem are identified. In this regard, a core microbiome represents those genomes or genetic markers common to the majority of samples considered. These are generally abundant in a given sample and have been hypothesized to mark important genetic functions and members of the microbial community ([10] and references therein). Such core microbiomes have been identified for different parts of the human body [11], plants [12], terrestrial systems [13], and marine ecosystems such as the pelagic realm [14] and methane seeps [15]. Using 454 tag sequencing, globally distributed marine sediment samples were also found to consist of characteristic bacterial classes, which were distinct from pelagic communities [6].

The surface deep-sea floor represents a rather uniform, specific environment, characterized by low temperatures (-1° to 4°C), high pressures (several hundred bars) as well as the absence of light and hence photosynthesis. Other key characteristics are a generally low supply of organic matter (1–10 mmol C m^-2^ yr^-1^), and fine-grained oxygenated sediments (250–300 μM oxygen) forming a dense sediment matrix of low permeability [3]. It is therefore likely that environmental selection has led to the establishment of a core deep-sea sediment microbiome that is distinct from those of other deep-sea environments. In support of this hypothesis, substantial differences in bacterial and archaeal communities of subsurface sediment, seep and vent ecosystems have been detected [15, 16]. But little is known about the community similarity of these seafloor systems to that of typical deep-sea sediments and of the spatial turnover of bacterial communities in deep-sea sediments. It may be assumed that the dimensions of the deep-sea realm are too large to support a global dispersal of sediment microorganisms, especially given the sluggish deep ocean currents.

Animal communities in the deep sea have been studied for a much longer time than microbial communities, and previous studies have shown a high degree of endemism for deep-sea animals, with most species only recorded as one or two individuals from one or two sampling sites [17]. Also for microbial eukaryotes, most taxa seem to be regionally restricted, with only few maintaining cosmopolitan distributions, and indicating positive range-abundance relationships (i.e. the size of the species geographic range increases with species abundance) [18]. Positive range-abundance relationships for bacterial populations have been observed in a variety of other microbial realms, including soil and the pelagic realm [19–24] and have originally been described for macroorganisms [25, 26]. But for deep-sea bacterial communities, biogeographic patterns, such as endemism vs. cosmopolitanism or species range-abundance relationships, remain largely unknown. In a first analysis at the global scale, indications of high levels of provincialism were found for bacterial communities in marine sediments [6], suggesting a limited dispersal of marine benthic bacterial communities in the deep sea. Also previous regional studies of deep-sea sediment bacterial communities (e.g. [27–32]) found a high degree of endemism and a high turnover of bacterial communities on the scale of meters to kilometres. However, all of these studies were regionally restricted or only marginally touched upon deep-sea sediments, and did not specifically test biogeographic patterns across the vast oligotrophic bathyal and abyssal seafloor.

Here, we used a dataset of 27 deep-sea surface sediment samples from all major ocean regions (top 1–2 centimetres of sediment), which focused on the zone between 1000–5300 m water depth, representative of 70% of the depth distribution of the global deep-sea floor [33]. We investigated four ecological rules, namely that I) The deep-sea surface sediment microbiome is distinct from subsurface communities. II) It is composed of a few sequence-abundant types, which form a core microbiome, and of many rare, endemic types. III) There is a positive relationship between the sequence-abundance of a taxon and the size of its geographic range (i.e. positive range-abundance relationship). IV) Deep-sea sediment bacterial communities exhibit a distance-decay relationship, i.e. community similarity decreases with increasing geographic distance due to isolation by distance and lack of population mixing.

## Material and Methods

### Datasets and 454 massively parallel tag sequencing (454 MPTS)

Deep-sea sediment samples analysed here mainly originated from our own sample repository (n = 20; S1 Table), but also included data from the ICoMM initiative (n = 7) [34], i.e. from the South Pacific (NZS) and the North Pacific (SMS). The 27 samples were obtained from water depths between 1025 m and 5347 m by winch-operated coring with a multiple corer, and consisted of 0.5–1 g of the top layer (0–2 cm) of deep-sea sediments composed of fine clays and biogenic particles. Sediment subsamples were removed directly after retrieval with a clean spatula and stored into cryovials at -20°C until DNA extraction. A list of all samples used in this study, their corresponding project names and geographic locations can be found in the Supporting Information (S1 Table). Permits for coring seafloor sediments within the Exclusive Economic Zones of coastal states were acquired before each seagoing expedition (S1 Table) from the legal authorities where necessary. The locations sampled are not privately-owned or protected in any way and the field studies did not involve endangered or protected species.

In all cases, sequencing data of the V6 region of the bacterial 16S rRNA gene were obtained according to the standardized sequencing pipeline of the ICoMM project (see S2 Table for the primer cocktail used; https://vamps.mbl.edu/index.php) [35, 36]. Fragments were sequenced by pyrosequencing on a Genome Sequencer FLX system (Roche, Basel, Switzerland) at the Marine Biological Laboratory in Woods Hole, MA, USA. Standard flowgram format files (sff) have been deposited in the GenBank Sequence Read Archives (www.ncbi.nlm.nih.gov/sra) and their accession numbers are provided in the Supporting Information (S1 Table). Flowgrams were processed and converted into an OTU-by-sample table with *mothur* (Version 1.29.2) [37] according to the standard operating procedure [38], including the implemented denoising algorithm [39]. Sequences were clustered into operational taxonomic units at a 3% nucleotide difference (hereafter referred to as OTU_0.03_). Alignment of sequences and taxonomic classifications were carried out using the SILVA reference database (release 119) [40] and the *mothur* standard operating procedure.

The global deep-sea surface sediment dataset comprised in total 501,480 sequences, corresponding to 88,247 OTU_0.03_. Absolute singletons (SSO_abs_), i.e. OTU_0.03_ consisting of a sequence occurring only once in the full dataset [41], accounted for 63% of all OTU_0.03_ (11% of all sequences). A reduced dataset with absolute singletons excluded hence comprised 455,822 sequences and 32,589 OTU_0.03_. We also defined a group of relative singletons (SSO_rel_) as those OTU_0.03,_ that were found in several samples of this global study, but occurred at least once as singleton, i.e. with one sequence in at least one sample [41]. This group accounted additionally for 19% of all OTU_0.03_ (3% of all sequences). The relevance of such rare types in bacterial communities is not well resolved in general [42–44], and may indicate either that bacterial diversity is still under-sampled, or that the observed diversity is the result of technical artefacts (i.e. PCR or sequencing errors). With the systematic noise-removal that we applied here to all datasets using the *mothur*-implemented denoising algorithm, technical (i.e. sequencing) errors are likely to be greatly reduced in our study. Earlier studies have also suggested that the removal of singletons or rare types neither affect the overall patterns of bacterial communities nor their ecological interpretation [6, 45, 46]. However, to account for any uncertainties related to the presence of SSO_abs_, we report results based on a reduced dataset with SSO_abs_ excluded, unless indicated otherwise.

### Potential contaminants in sequencing data

Betaproteobacteria, especially affiliated to *Burkholderiales* and *Ralstonia*, have been reported from deep-sea sediments and also for the terrestrial deep subsurface ([16] and references therein). However, there are also indications that these sequences may originate from contamination of clean laboratory water or reagents [47–50]. In our current dataset, OTU_0.03_ affiliated with *Burkholderiales* accounted for 5% of Betaproteobacteria OTU_0.03_ (36% of Betaproteobacteria sequences). None of these OTU_0.03_ occurred in all 27 samples (e.g. only 3 OTU_0.03_ occurred in 21 and 20 samples), while contaminant sequences would have been expected to occur in all samples. A contamination with sequences from laboratory reagents would need to be tested individually for each protocol, sample analysis pipeline and reagents used, and would need to be conducted in parallel with sample handling. We therefore could not categorize specific sequences as contaminants based on sequence similarities to previously found contaminants, and refrained from excluding sequences a priori from this dataset.

### Statistical analyses

Observed richness (i.e. number of OTU_0.03_ per sample) and richness estimates (Chao1) were calculated with 100 sequence re-samplings per sample based on the smallest dataset (n = 7,922 and 6,883 sequences with and without SSOabs respectively), to account for differences in sequencing depth between samples. Overall differences in bacterial community composition were visualized with non-metric multidimensional scaling plots. A corresponding analysis of similarity was used to assess significant differences between samples grouped by oceanic regions. Shared or endemic OTU_0.03_ (i.e. found in only one sample or oceanic region) were also calculated with 100 sequence re-samplings per sample based on the smallest dataset. Geographic distances between stations were calculated in two different ways: i) as the surface distance between samples (function *geodist* in R package ‘gmt’), and ii) as the shortest path by sea between samples, only allowing connecting routes through water (function *lc*.*dist* in R package ‘marmap’). To test whether community similarity was significantly correlated with different spatial components, non-parametric Mantel tests [51, 52] based on the Spearman correlation coefficient were applied and significance assessed based on 1000 Monte Carlo permutations.

We further tested how biogeochemical provinces defined by Longhurst et al. [53] (http://www.vliz.be/vmdcdata/vlimar/downloads.php), and oceanographic regions based on total organic carbon measurements [54], as proxies for surface productivity and sediment total organic carbon content, accounted for changes in bacterial community similarity. A partitioning of the variation in bacterial community composition between spatial distance, water depth, surface productivity and total organic carbon was conducted according to Legendre [55]. All statistical analyses were performed in R (v. 3.1.1) (R Development Core Team 2009, http://www.R-project.org) using packages *vegan* [56], *gplots* [57], *gmt* [58], *marmap* [59], *gdistance* [60] and with custom R scripts that are provided in the supplementary information (S1 Script).

## Results & Discussion

Recent studies found deep-sea sediments to be extremely rich and diverse in bacterial types despite the low quantities of organic matter available, rivalling even the diversity of much more organic rich soils on land [61]. The deep-sea floor samples analysed here exhibited an average Chao1 richness estimate of 4,599 ± 1572, with absolute singletons included (for Chao1 richness estimates with and without SSOabs see S1 Fig). However, since the Chao1 estimator corrects the observed richness by adding a term based on the number of singletons and doubletons, we also report here the observed richness. Observed richness was on average 2,623 ± 554, ranging from 2,245 ± 267 (average for South Atlantic) to 3,499 ± 416 (average for South Pacific) (S2 Fig). These estimates are within the range of, or higher than values reported from regional studies of deep-sea sediments [31, 32]. They are substantially higher than estimates from the ocean pelagic realm [14, 62], seeps and vents [15]. Accumulation curves (S3 Fig) indicated that at coarse taxonomic resolution (i.e. phylum to family), the diversity of most taxa was captured with this global sample set, but a considerable part of the global bacterial diversity in deep-sea sediments at the OTU_0.03_ level was still missing.

### The microbiome of deep-sea surface sediments

**Fig 1:**
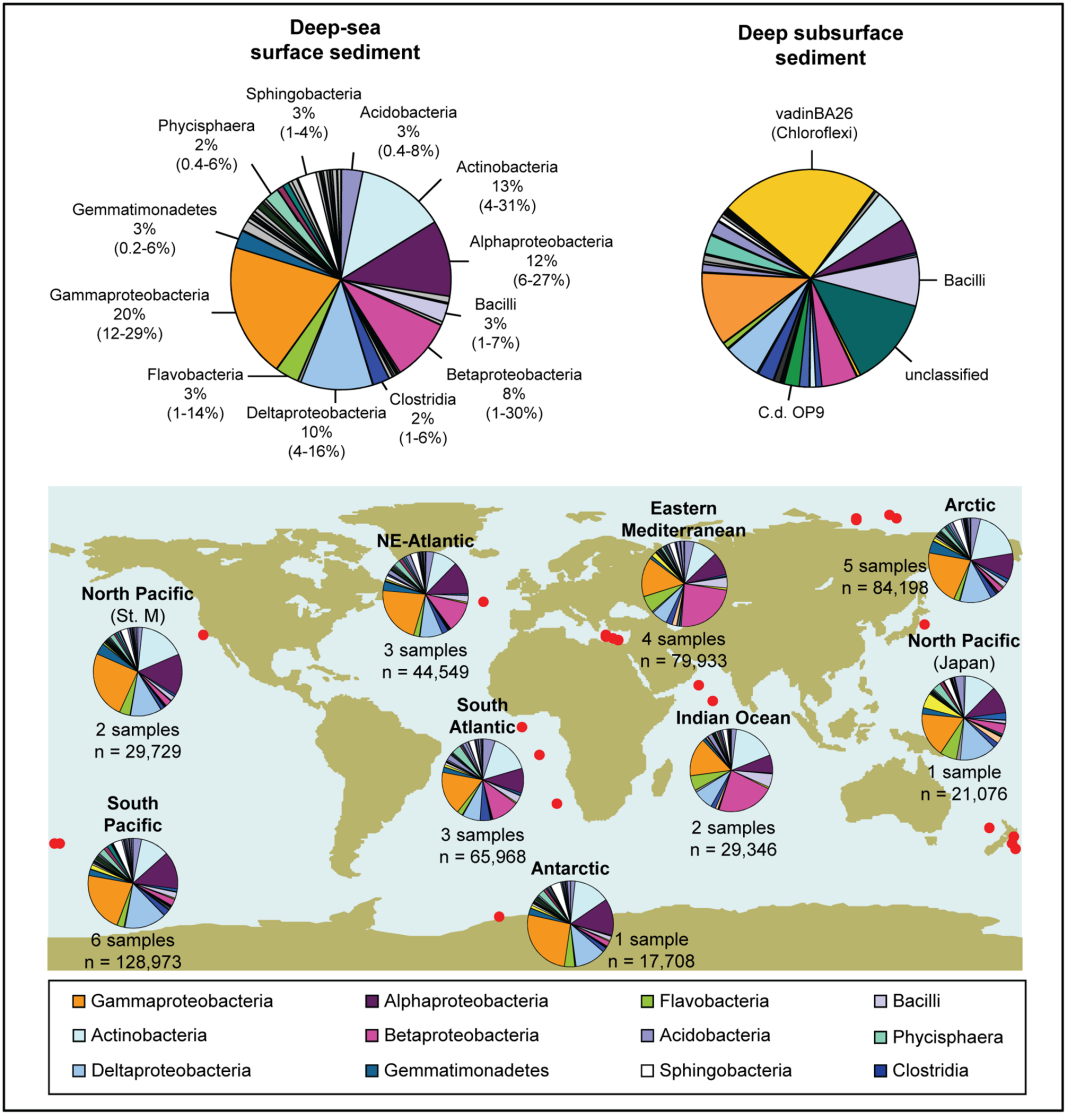
Community composition of bacterial communities in deep-sea sediment (water depth ≥ 1000 m), at the class level (89 classes). The large pie chart (top left) summarizes the findings based on all samples (N = 27 samples), and indicates the average relative abundances (only when ≥ 2%) of each class and the associated ranges in individual samples. Small pie charts on the map give the average community compositions in nine different oceanic regions. The numbers of samples as well as the number of sequences (n) are indicated. For comparison, the average community composition in subsurface sediments (2.5–90 mbsf, N = 5 samples, 98 classes) (http://icomm.mbl.edu, projects ICM_CFU and KCK_ODP) is displayed (top right). All sequence data were denoised and analysed using the standard operating procedure in *mothur*.

Marine sediments characteristically show a dominance of *Proteobacteria* [6, 34]. Also in this global study of deep-sea sediments, half of the sequences belonged to the phylum *Proteobacteria* (50%), most of which were affiliated with the classes *Gammaproteobacteria (*20 ± 5%), *Alphaproteobacteria* (12 ± 4%), and *Deltaproteobacteria* (10 ± 4%) (Fig 1). The phylum *Actinobacteria* was second in sequence abundance (13 ± 6%). *Gammaproteobacteria* sequences were the most abundant at the majority of sampling sites. The dominance of these taxonomic groups is in agreement with both global [6] and regional studies, e.g. in the Eastern Mediterranean [63], Arctic [28, 29], East Pacific [27], South Pacific [64], and South Atlantic Ocean [30], the latter being based on 16S rRNA gene clone libraries. The deep-sea surface sediment community differs from surface and deep-water communities, which are usually dominated by *Alphaproteobacteria*, *Gammaproteobacteria*, *Cyanobacteria* and *Flavobacteria* (e.g. [6, 14, 65]).

We further compared the average composition of deep-sea surface sediment communities to an average community from subsurface sediment (5 samples between 2.5 and 90 m below seafloor from the Peru Margin), which were analysed with the same sequencing and bioinformatics methods (454 pyrosequencing of the V6 region; see http://icomm.mbl.edu) (Fig 1, S2 Table). The deep-sea surface sediment community differed clearly from subsurface sediments in the dominance of *Gammaproteobacteria*, and in the rarity of *Chloroflexi* (vadinBA26), *Bacilli* and candidate division OP9/JS1 that are typical of subsurface environments [16, 66–69]. This demonstrates that pelagic, benthic surface and deep-subsurface environments exhibit distinct bacterial community signatures already at broad taxonomic resolution levels. At a finer taxonomic resolution, even less overlap was detected, with none of the twenty most abundant OTU_0.03_ from each environment shared between the two realms (S3 Table), probably reflecting differences in life styles and environmental pressures between these habitats, even though this comparison is yet based on a limited number of samples.

The deep-sea sediment core microbiome, here defined as OTU_0.03_ that occurred in more than 90% of the deep-sea surface sediment samples (i.e. in ≥ 25 of the 27 samples) and in all oceanic regions, consisted of only 18 OTU_0.03_ (0.1% of all OTU_0.03_), comprising 6.2% of all sequences (Table 1). They included many taxa comprising heterotrophic polymer degraders, as is expected for deep-sea sediment communities, where the main source of energy and nutrients is marine detritus [3]. Only three highly abundant OTU_0.03_ were truly cosmopolitan (i.e. found in all 27 samples). Among these, two OTU_0.03_ were affiliated with the JTB255 clade (order *Xanthomonadales*), and one with the OM1 clade (order *Acidimicrobiales*) (Table 1, S3 Table); these OTU_0.03_ made up 21% of the JTB255 and 7% of the OM1 clade. Nevertheless, the truly cosmopolitan OTU_0.03_ of the JTB255 and the OM1 clade also showed variations in relative sequence abundance (in relation to the total number of sequences per sample) between oceanic regions. They ranged from 0.1% in the Eastern Mediterranean to 3.6% in the Antarctic for JTB255, and from 0.1% in the Eastern Mediterranean to 1.4% in the Arctic Ocean for OM1 (S4 Fig). Thus, both types appear to be more sequence-abundant in polar, cold regions (e.g. Antarctic, Arctic), and less abundant in warmer regions (e.g. Mediterranean). Overall, sequences of the JTB255 clade have been reported in a range of local and regional marine benthic studies (e.g. [28, 30, 70–72]), but the function of this group remains unknown, as no relative has yet been cultivated. Previously, members of the OM1 clade have been predominantly described from seawater [73, 74], and further investigations are needed to address their functional relevance in deep-sea sediments.

**Table 1:** Most common OTU_0.03_ occurring in 90% of the samples (i.e. in ≥25 out of 27 samples), their taxonomic affiliation, and their sequence abundance in this global set of deep-sea surface sediment samples.

Class	Order	Family	Genus	Number of samples in which present	Absolute sequence abundance in dataset	% sequence abundance in whole dataset	% sequence abundance across oceanic regions		
							Average	Min.	Max.
Gammaproteobacteria	Xanthomonadales	JTB255_marine_benthic_group	unclassified	27	4513	1.01	1.16	0.07	2.76
Acidimicrobiia	Acidimicrobiales	OM1_clade	unclassified	27	2399	0.54	0.58	0.05	1.19
Gammaproteobacteria	Xanthomonadales	JTB255_marine_benthic_group	unclassified	27	1963	0.44	0.36	0.08	0.90
Acidimicrobiia	Acidimicrobiales	OM1_clade	unclassified	26	4226	0.95	1.00	0.06	2.66
Gammaproteobacteria	Xanthomonadales	JTB255_marine_benthic_group	unclassified	26	3611	0.81	0.68	0.12	1.94
Gammaproteobacteria	Xanthomonadales	JTB255_marine_benthic_group	unclassified	26	2316	0.52	0.60	0.06	1.48
Gemmatimonadetes	BD2-11_terrestrial_group	unclassified	unclassified	26	1084	0.24	0.24	0.07	0.47
Gemmatimonadetes	BD2-11_terrestrial_group	unclassified	unclassified	26	594	0.13	0.14	0.06	0.23
JTB23	unclassified	unclassified	unclassified	26	903	0.20	0.24	0.05	0.58
SPOTSOCT00m83	unclassified	unclassified	unclassified	26	624	0.14	0.11	0.02	0.26
Alphaproteobacteria	4-Org1-14	unclassified	unclassified	26	270	0.06	0.08	0.02	0.19
Gammaproteobacteria	Gammaproteobacteria_Incertae_Sedis	Unknown_Family	Methylonatrum	25	973	0.22	0.22	0.03	0.40
Flavobacteriia	Flavobacteriales	Flavobacteriaceae	Aestuariibaculum	25	1596	0.36	0.60	0.07	3.28
Gammaproteobacteria	BD7-8_marine_group	unclassified	unclassified	25	719	0.16	0.19	0.01	0.43
Gammaproteobacteria	Gammaproteobacteria_Incertae_Sedis	Unknown_Family	Methylonatrum	25	520	0.12	0.11	0.01	0.19
Gammaproteobacteria	Order_Incertae_Sedis	Family_Incertae_Sedis	Marinicella	25	761	0.17	0.17	0.02	0.43
Gammaproteobacteria	Methylococcales	pItb-vmat-59	unclassified	25	404	0.09	0.12	0.03	0.26
Gammaproteobacteria	Methylococcales	pItb-vmat-59	unclassified	25	248	0.06	0.07	0.02	0.16

Our study supports the hypothesis of a distinct core microbiome in global deep-sea sediments with yet unknown adaptations, as no close relative has been cultivated yet or has had its genomic composition established. This core bacterial community may consist of generalists highly adapted to life in the deep sea, e.g. with a high flexibility in the use of resources, similar to what has been suggested for the few cosmopolitan types in soil microbial communities [75]. In addition, we tested whether the diversification of this core microbiome accounts for a substantial fraction of the observed diversity of surface deep-sea sediments, as shown for the microbiome of cold seeps [15]. In cold seep communities, endemic taxa closely related to the members of the core microbiome make up a substantial proportion of total richness. This trend could not be confirmed for deep-sea sediments, as those families that contained the 18 most abundant OTU_0.03_ contributed only a small proportion of the endemic types. Our data suggest that a substantial fraction of the global diversity of bacteria in deep-sea sediments is endemic.

### Deep-sea sediment bacteria endemism, cosmopolitanism and positive range-abundance relationship

Since deep-sea sediments can be considered as a relatively stable and uniform environment, forming a matrix of fine particles that immobilizes their bacterial inhabitants, dispersal of benthic bacteria in the deep sea is probably limited. Microbial dispersal may, however, occur via the resuspension of sediments by water currents and faunal activity (i.e. bioturbation or by bentho-pelagic organisms feeding on sediments and migrating (e.g. [76, 77]). Yet, deep-water currents above the seafloor are usually weak, hence long-distance passive transport of deep-sea sediments probably occurs rarely [78, 79].

**Fig 2:**
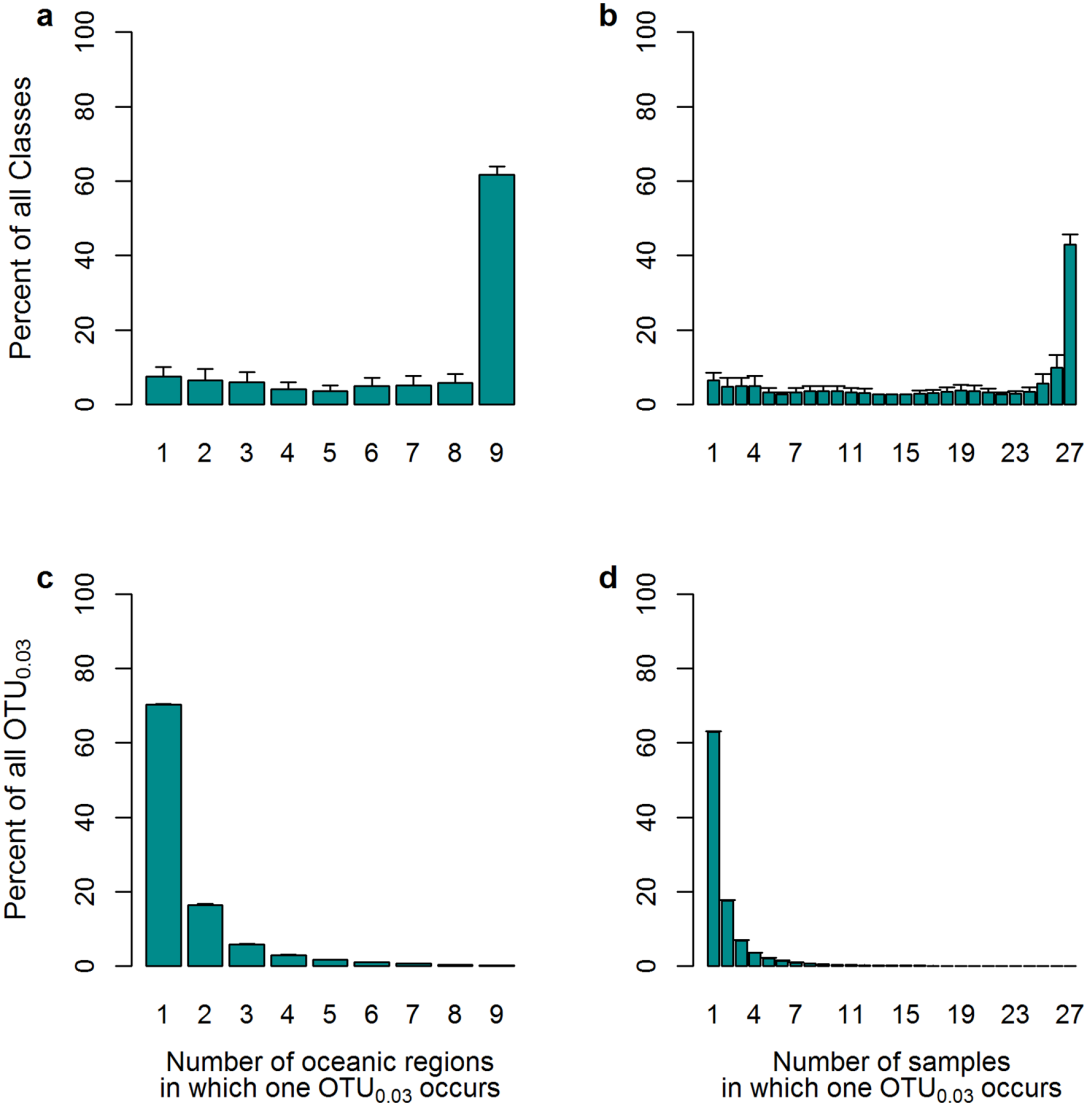
Proportions of unique and cosmopolitan OTU between oceanic regions and individual samples at the class (a, b) and OTU_0.03_ (c, d) level, after averaging of 100 sequence random resampling results (n sequences = 6883, standard deviations are indicated).

Comparing community composition at the broad taxonomic levels of phylum to class, a rather uniform distribution of the sediment microbiota was detected across all oceans (Fig 2a–2b, S5 Fig). Differences in community composition appeared at the family and higher taxonomic resolution levels (data not shown), as reported from other global microbiome studies of permafrost soils [80], cryosphere habitats [81], or other environments (see also [46]). Previous studies of bacterial OTU-distribution have shown that average spatial ranges of OTU can change with environment, latitude and sequence abundance [23, 82]. Here, a high degree of endemism was detected; higher than in water column environments [82]. At the resolution of OTU_0.03_, up to 70% of all bacterial taxa were unique to one sample (Fig 2c and 2d), and the proportion of pairwise shared OTU_0.03_ between oceanic regions was 11% on average (ranging from 3–19%; S6 Fig). Absolute singletons (SSO_abs_) logically increased the levels of endemism at the OTU_0.03_ level (to ca. 80%, S7 Fig). This observation supports previous findings on global bacterial distribution in other ocean realms [23, 82]. In comparison, the Census of the Diversity of Abyssal Marine Life also reported that the majority of deep-sea animals occurred at only one or two sampling sites, at a similar proportion as bacterial taxa in this study [17]. However, it is not known whether this recurring observation points to severe undersampling of the deep sea, or indeed to rarity and limited dispersal [17, 83].

**Fig 3:**
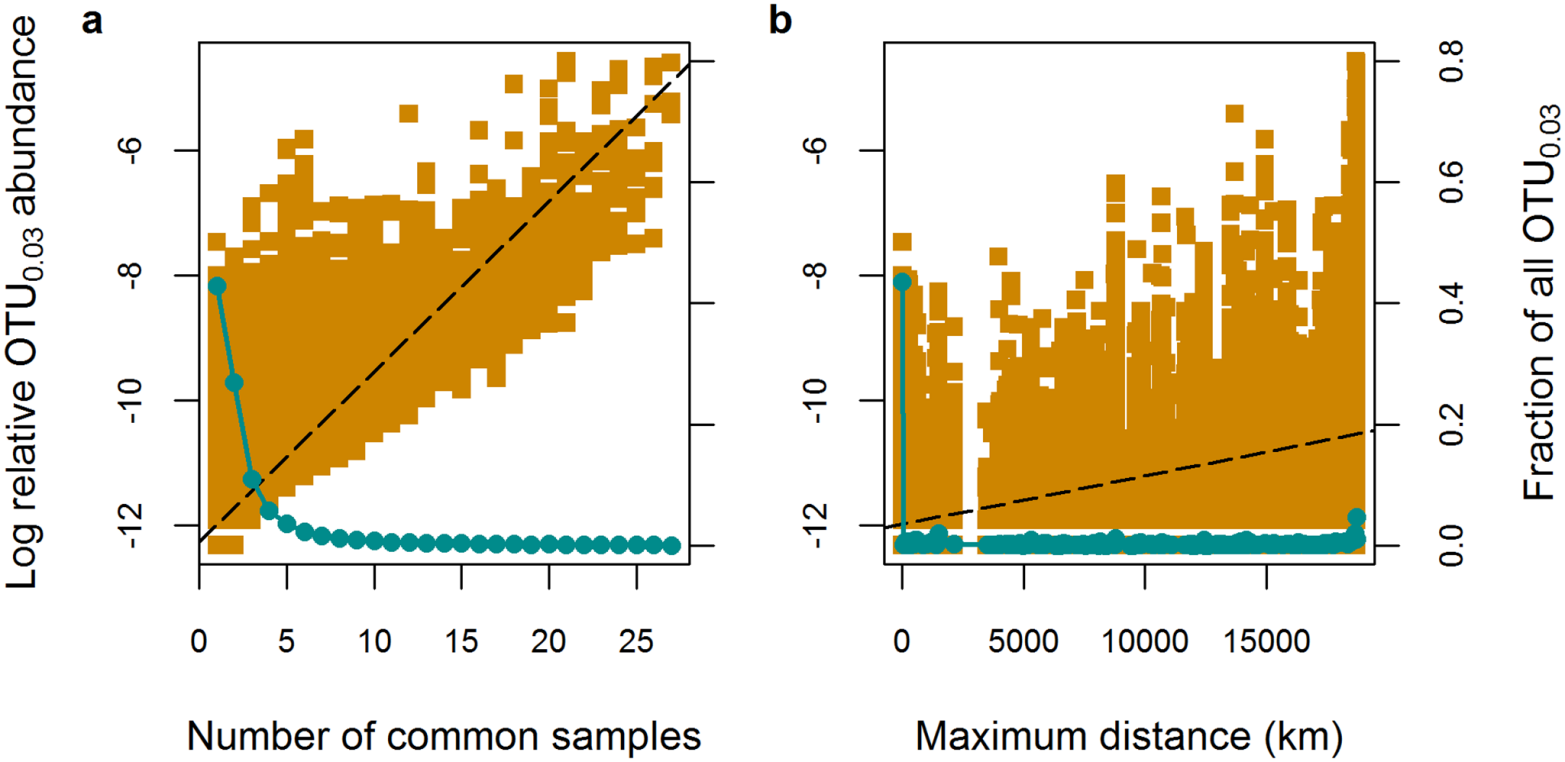
Range-abundance relationships. **a)** Log-transformed relative OTU_0.03_ sequence abundance (filled orange squares) as a function of the number of samples an OTU_0.03_ was detected in, and the fraction of OTU_0.03_ from the total number of OTU_0.03_ (filled blue circles) that fall into the different categories. **b)** Log-transformed relative OTU_0.03_ sequence abundance (filled orange squares) as a function of the maximum distance an OTU_0.03_ was detected at, and the fraction of OTU_0.03_ from the total number of OTU_0.03_ (filled blue circles) that fall into the different range classes. Dashed lines indicate linear models for range-abundance relationships: a) Adj. R^2^ = 0.66, p<0.0001, b) Adj. R^2^ = 0.30, p<0.0001.

We looked at another group of rare OTU_0.03_, i.e. those that occurred in three samples or less (≤10% of all samples). These OTU_0.03_ were of low sequence-abundance (<0.01% of all sequences) and accounted for 80% of all OTU_0.03_, therefore they displayed the typical long tail of rare types observed in microbial rank-abundance curves [36, 84] (Fig 3a). The majority of these OTU_0.03_ had an average geographic range of 3,781 km (ranging from 0–18,700 km), suggesting that most low abundance OTU_0.03_ are limited in their range to within ocean basins (Fig 3b). Such a high turnover of OTU_0.03_ between samples has also been evidenced in other studies at smaller spatial scales (tens to hundreds of kilometres) in Arctic deep-sea sediments [31], for marine bacterioplankton communities [21], or when considering different habitat types [20]. In contrast, sequence-abundant OTU_0.03_ (defined as OTU_0.03_ comprising >0.1% of all sequences) were found across average distances of 18,029 km (ranging from 8,000–18,700 km). Consequently, the more sequence-abundant an OTU is, the more likely it is to be found in samples located much further away. These results indicate that, despite our assumption of very slow rates of dispersal in the deep-sea environment, it is still possible to observe truly ubiquitous, cosmopolitan taxa, at the level of their 16S (V6) gene signature. Future studies should direct effort to the question of their identities, traits and functions, to better understand the evolution of core microbiota in the deep-sea realm and on Earth in general.

The analysis of how relative sequence abundance changes with geographic range (either defined as the number of common samples or the maximum distance an OTU_0.03_ is observed at) supported the presence of a positive range-abundance relationship (Fig 3). Comparable patterns have been reported for microbial eukaryotes in the deep sea, where the majority of taxa were regionally restricted, and only a small percentage maintained cosmopolitan distributions [18]. Positive range-abundance relationships for bacterial types have also been observed in a variety of other microbial realms, including soil and the pelagic realm [19–24].

While methods based on sequencing of 16S rRNA genes do not fully reflect the true abundance of organisms in the environment [85], a plausible ecological explanation for the observed positive range-abundance relationship would be that higher local population sizes—as approximated by high sequence abundances here—enable a larger organismal pool to be further passively dispersed, higher colonization and lower extinction rates (mass-effect of metapopulation dynamics as described in e.g. [86]). In addition other mechanisms may generate positive range-abundance relationships, such as resource breadth and availability, also proposed previously [86, 87]. As aforementioned, undersampling is most likely one reason for the pattern observed here, as we still miss—despite the use of high-throughput sequencing—very low abundant types in some samples (S3 Fig) and therefore underestimate their distribution ranges.

### Distance-decay and predictors for variation in bacterial surface sediment communities

**Fig 4:**
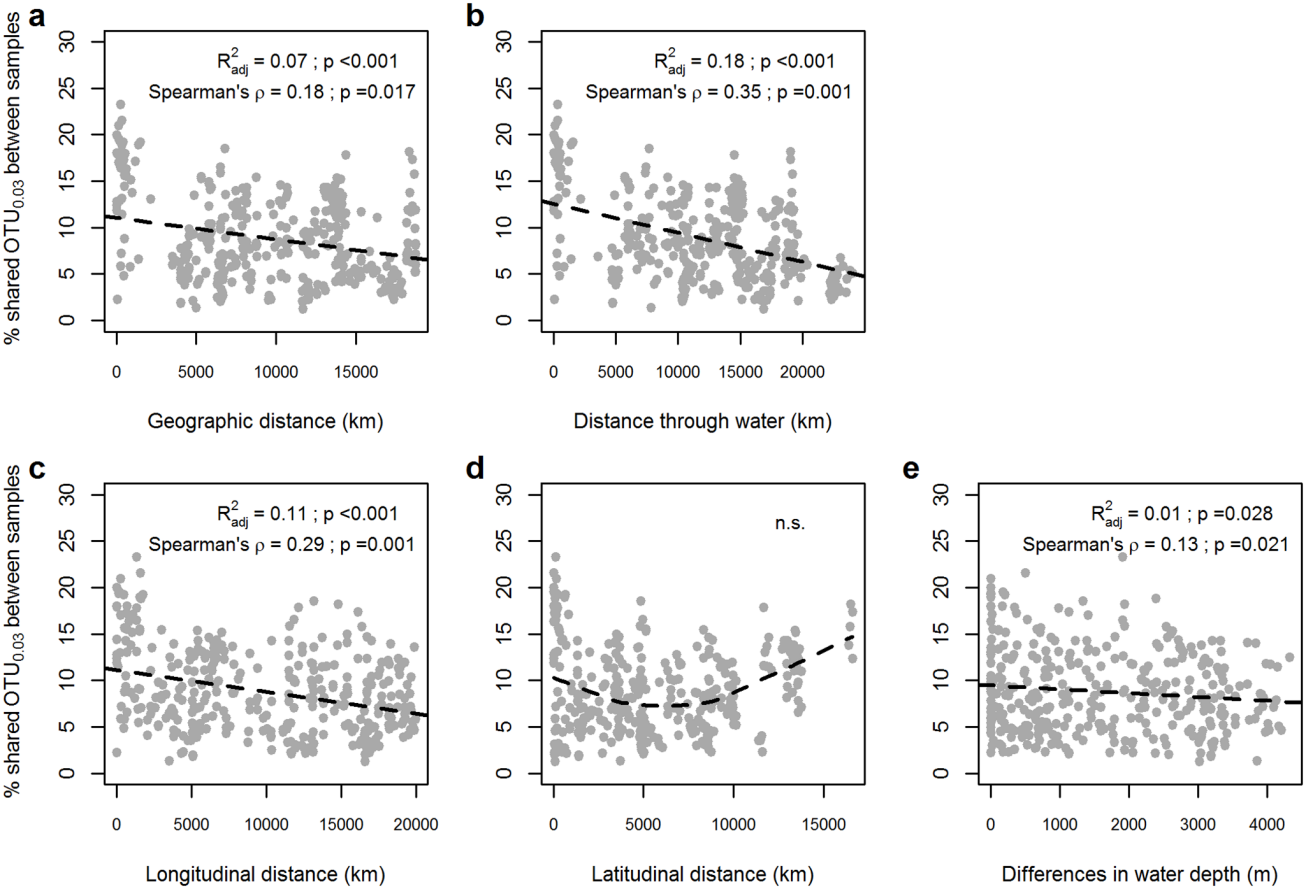
Distance-decay and geographic patterns of bacterial deep-sea sediment communities. The proportion of shared OTU_0.03_ between samples significantly decreased with geographic (earth surface) distance (**a**) and with distance through water (**b**). The proportion of shared OTU_0.03_ decreased with longitudinal distance (**c**), showed no correlation with latitudinal distance (**d**), and correlated with water depth (**e**). Dotted lines are linear model fits. Linear model’s R^2^, Spearman’s rho correlations, and their significance (Mantel tests with 1000 permutations) are reported in each panel (n.s., not significant). The dotted line in **d** displays a LOESS curve to indicate the trend with latitudinal distance.

Significant distance-decay relationships for bacterial communities have been reported in a global study of pelagic and seafloor environments [82], in soil [88–90], woodland [91], and saltmarsh sediments [92], suggesting this relationship to hold true across different ecosystems. Here we focused on deep-sea sediments at >1000 m water depth. We found that community similarity (based on the proportion of shared OTU_0.03_) between samples decreased significantly with increasing geographic distance (Fig 4a), with a slope coefficient for the distance-decay relationship |ß| in our dataset of 0.066 (calculated accordingly to [82]).

We also tested whether the distance-decay relationship holds true when considering water paths around continents instead of straight distances (earth surface) between sampling locations (Fig 4b). The explained variation and slope of the relationship (|ß| = 0.088) were higher than when considering direct connecting lines, reinforcing the idea of spatial isolation in deep-sea bacterial communities, when using an appropriate distance metric. A connectivity of microbial populations via deep-water currents has been suggested for sediment and deep-water communities, and for benthic thermophilic endospores [93–95]. This dispersal mechanism would be similar to what has been proposed for larval stages of benthic deep-sea fauna [1]. In the future, more advanced sampling schemes and models [96] should be applied to test for the effect of deep-water transport (speed, direction) on bacterial deep-sea communities.

The distance-decay relationship observed for bacterial communities may arise from multiple mechanisms, involving environmental filtering, neutral processes, and isolation by distance, which is a complex product of limited dispersal, ecological drift, and speciation processes [97]. To shed more light on the mechanisms generating the observed distance-decay relationship, we also considered changes in community similarity as a function of differences in water depth, latitudinal distance, and longitudinal distance (Fig 4c–4e). Increasing water depth is a general indicator of decreasing particle flux as key energy source for deep-sea bacteria [3]. A relatively weak, but significant relationship was observed for community changes along water depth (Fig 4). This confirmed that even below 1000 m, bacterial communities are structured by changes in biological or physical parameters that are correlated with water depth, especially the dynamics in particulate organic matter flux that represent the main source of energy and carbon [31, 98]. The range of investigated water depths itself did not explain a significant fraction of community variation when other variables, such as geographic distance or organic carbon content, were considered (S8 Fig).

Latitudinal distance correlates with climatic regions of the surface ocean, and previous studies have reported correlations between bacterial community richness and latitude for communities from the pelagic [21, 99], and from terrestrial realms [100, 101] (for controversial findings see [93, 102]). But, according to the physical stability of the deep sea, latitudinal distances were neither a good predictor of community similarity (Fig 4d), nor of richness in deep-sea surface sediment communities (S9 Fig). However, a trend analysis based on LOESS curve fitting (Fig 4d) indicated that community similarity increased towards both polar regions, as detected already for epipelagic marine bacteria [24]. This may be related to latitudinal changes in ocean productivity and particle flux, which increase both southwards and northwards from the equator to about 70° latitude in both hemispheres [53]. Distances along latitude and longitude were also correlated with water depth (Spearman’s rho = 0.2 and 0.28, p = 0.01 and 0.006 for latitude and longitude, respectively.) But interestingly, changes in community similarity with geographic distance appeared to be mainly due to changes with longitude (Fig 4c). On the one hand, geographic features like mid-ocean ridges, and deep-water currents [83], but also land masses, may present barriers to dispersal along longitudinal axes. However, this pattern may also result from changes in productivity regimes with proximity to the productive ocean margins.

### Effects of spatial and environmental parameters on seafloor bacterial community composition

We further tested how other environmental parameters may account for changes in bacterial community composition (based on relative sequence abundances) with geographic distance. For example, the role of surface productivity, particle flux, and of other biological factors in the structuring of benthic communities have previously been suggested [6, 98]. Productivity indices based on biogeochemical provinces defined by Longhurst et al. [53] (http://www.vliz.be/vmdcdata/vlimar/downloads.php) and oceanographic regions based on total organic carbon measurements [54] were used as proxies to estimate environmental differences at the global scale. A partitioning of the biological variation between geographic distance, water depth, surface productivity and total organic carbon content confirmed a significant effect of geographic distance (4% of variation explained, p = 0.04) on bacterial community structure, even when taking other environmental parameters into account (S8 Fig). However, differences in total organic carbon at the seafloor also played a significant role in shaping bacterial community structure (10% of variation explained, p = 0.003), and in addition there was a noticeable level of co-variation between geographic distance and total organic carbon categories (3%). Also surface productivity explained some of seafloor bacterial community variation, but this was not statistically significant (3% of variation explained, p = 0.058). Discrepancies between surface productivity and total organic carbon availability at the seafloor may be explained by biological processes or hydrographic features altering vertical particle flux, or by a lateral input of organic material. The effect of organic matter availability on benthic communities is in agreement with general trends reported for different benthic size classes in the deep sea [1, 98, 103–105]. The effects of these factors on bacterial community structure and distribution will need to be further explored for the deep seafloor at the global scale. Future studies should aim at integrating different spatial scales and at measuring a large range of environmental parameters, e.g. total organic carbon, particle flux, nutrients, chlorophyll pigments, as well as biological factors such as the presence of fauna, to provide potential descriptors of microbial community patterns in the deep sea.

## Conclusion

By investigating the composition and distribution of benthic deep-sea bacterial communities at the global scale, we show that bacterial communities of deep-sea surface sediments are distinct from those of the pelagic or the subsurface seafloor biosphere, and this already at the class level. Deep-sea sediments are inhabited by a core community of few cosmopolitan, sequence-abundant bacterial OTU which are affiliated with the JTB255 marine benthic group (class *Gammaproteobacteria*, order *Xanthomonadales*), and the OM1 clade (class *Actinobacteria*, order *Acidimicrobiales*), but which still lack representative genomes and cultured organisms. At the same time, our study revealed a high degree of endemism and isolation, hence a significant part of bacterial communities in deep-sea surface sediments appears to be geographically restricted. We found evidence that the relative sequence-abundance of a taxon and the size of its geographic range are positively related to each other. We also detected that deep-sea sediment bacterial community similarity decreases with increasing geographic distance, most likely due to isolation-by-distance processes (especially along longitudes). Bacterial communities mostly changed with indicators of productivity regimes, such as TOC content of sediments.

## Supporting Information

S1 Fig Chao1 richness estimates (blue, left y axis) calculated with 100 sequence re-samplings for data without (a) and with (b) SSOabs (n resampling = 6,883 and 7,922 sequences for a and b, respectively). Standard deviations for richness are indicated in black. Water depth of each sample is displayed in red (right y axis). No significant relationship was found between richness and water depth (Spearman’s ρ = -0.33 and -0.37 for a and b, respectively, *P* > 0.05 in both cases).
(PDF)

S2 Fig Observed richness (blue, left y axis) calculated with 100 sequence re-samplings for data without (a) and with (b) SSOabs (n resampling = 6883 and 7922 sequences for a and b respectively). Standard deviations for richness are indicated in black. Water depth of each sample is displayed in red (right y axis). The relationship between richness and water depth was significant but weak (Spearman’s ρ = -0.44 and -0.43; *P* = 0.02 in both cases).
(PDF)

S3 Fig Species accumulation curves based on different bacterial taxonomic categories: phylum to family (a), genera (b), and OTU_0.03_ level (c). Colors in **a**) mark the taxonomic categories phylum: white, class: red, order: orange, family: yellow. The boxplots show a summary of 100 permutations, calculated with random subsampling, including absolute singletons for comparison.
(PDF)

S4 Fig Variations of truly cosmopolitan OTUs affiliated with the clades JTB255 (a, class *Gammaproteobacteria*, n = 2) and OM1 (b, class *Actinobacteria*, n = 1) between oceanic regions. Relative abundances were averaged across samples and oceans. Error bars indicate standard deviations when considering samples from one oceanic region.
(PDF)

S5 Fig Differences in bacterial community composition between oceanic regions. Non-metric multidimensional scaling plots for community composition at the class (**a-b**) and OTU_0.03_ (**c-d**) levels in terms of presence/absence (a,c; using the Jaccard index) and relative abundance (b,d; using the Bray-Curtis index). Samples originating from a same oceanic region are connected by a coloured line, as follows: black: South Pacific; red: North Pacific (St. M); green: Indian Ocean; blue: NE-Atlantic; light-blue: E-Mediterranean; pink: Arctic; yellow: North Pacific (Japan); brown: Antarctic; orange: South Atlantic. Differences in community composition were weak at the class level (ANOSIM R = 0.2 and 0.51, p = 0.03 and 0.001 for a and b, respectively), but significant at the OTU_0.03_ level (ANOSIM R = 0.7 and 0.66, p = 0.001 for c and d, respectively).
(PDF)

S6 Fig Percentage of shared OTU_0.03_ between oceanic regions. (PDF)

S7 Fig Proportions of unique and cosmopolitan OTU_0.03_ between oceanic regions and individual samples, including SSOabs, and after averaging of 100 sequence random resampling results (n sequences = 7,922, Standard deviations are indicated). (PDF)

S8 Fig Partitioning of the biological variation in bacterial community structure at the OTU_0.03_ level (with absolute singletons excluded) between the following contextual parameters: geographic distance between samples, water depth, TOC availability (TOC regions based on Seiter et al. 2004, Deep Sea Res., Part I 51: 2001–2026), and surface productivity (Longhurst productivity index based on Longhurst et al. 1995, J Plankton Res 17: 1245–1271). ** p = 0.01, * p = 0.05, (*) p = 0.07, as tested with 100 permutations.
(PDF)

S9 Fig Bacterial OTU_0.03_ richness as a function of latitude. Linear model is not significant.
(PDF)

S1 Table Contextual data for all deep-sea samples: VAMPS (http://vamps.mbl.edu) sample ID, geographic origin, water depth, oceanic region, and sequence archive accession numbers for GenBank Sequence Read Archives (www.ncbi.nlm.nih.gov). (PDF)

S2 Table Relative sequence abundance of the most abundant bacterial classes in deep water (>1000 m water depth), deep-sea surface sediment (>1000 m water depth), and deep subsurface sediment (2.5–90 m below seafloor) samples. (PDF)

S3 Table a) Twenty most abundant bacterial OTU_0.03_ for deep-sea surface sediment (> 1000 m water depth). Total number of samples considered is 27. Total number of sequences in the dataset is 501,480. b) Twenty most abundant bacterial OTU_0.03_ for deep subsurface samples (between 2.5 and 90 m below seafloor from the Peru Margin; http://icomm.mbl.edu). Total number of samples considered is 5. Total number of sequences in the dataset is 72,294.
(PDF)

S1 Script R script including all relevant analyses for this study. (R)

## References

[1] Rex MA, Etter RJ. Deep-sea biodiversity—pattern and scale Cambridge: Harvard University Press; 2010.

[2] Whitman WB, Coleman DC, Wiebe WJ. Prokaryotes—the unseen majority. Proc Natl Acad Sci U S A. 1998;95(12):6578–83. ZT829-0004. 9618454 10.1073/pnas.95.12.6578 PMC33863

[3] Jørgensen BB, Boetius A. Feast and famine—microbial life in the deep-sea bed. Nat Rev Microbiol. 2007;5(10):770–81. 2007-211LO-0011. 17828281 10.1038/nrmicro1745

[4] Boetius A, Wenzhofer F. Seafloor oxygen consumption fuelled by methane from cold seeps. Nature Geosci. 2013;6(9):725–34. 10.1038/ngeo1926

[5] Smith KL, Ruhl HA, Bett BJ, Billett DSM, Lampitt RS, Kaufmann RS. Climate, carbon cycling, and deep-ocean ecosystems. Proc Natl Acad Sci U S A. 2009;106(46):19211–8. 10.1073/pnas.0908322106 19901326 PMC2780780

[6] Zinger L, Amaral-Zetter LA, Fuhrman JA, Horner-Devine MC, Huse SM, Mark Welch D, et al Global patterns of bacterial beta-diversity in seafloor and seawater ecosystems PLoS ONE. 2011;6(9):e24570 10.1371/journal.pone.0024570 21931760 PMC3169623

[7] Glover AG, Smith CR. The deep-sea floor ecosystem: current status and prospects of anthropogenic change by the year 2025. Environ Conserv. 2003;30(3):219–41. 10.1017/s0376892903000225 WOS:000185873100002.

[8] Kimes NE, Callaghan AV, Aktas DF, Smith WL, Sunner J, Golding BT, et al Metagenomic analysis and metabolite profiling of deep-sea sediments from the Gulf of Mexico following the Deepwater Horizon oil spill. Frontiers in Microbiology. 2013;4 10.3389/fmicb.2013.00050 PMC3598227 23508965

[9] Mason OU, Scott NM, Gonzalez A, Robbins-Pianka A, Balum J, Kimbrel J, et al Metagenomics reveals sediment microbial community response to Deepwater Horizon oil spill. ISME J. 2014;8(7):1464–75. 10.1038/ismej.2013.254 24451203 PMC4069396

[10] Shade A, Handelsman J. Beyond the Venn diagram: the hunt for a core microbiome. Environ Microbiol. 2012;14(1):4–12. 10.1111/j.1462-2920.2011.02585.x 22004523

[11] Li K, Bihan M, Methé BA. Analyses of the Stability and Core Taxonomic Memberships of the Human Microbiome. PLoS ONE. 2013;8(5):e63139 10.1371/journal.pone.0063139 23671663 PMC3646044

[12] Vorholt JA. Microbial life in the phyllosphere. Nat Rev Micro. 2012;10(12):828–40. 10.1038/nrmicro2910 23154261

[13] Gilbert JA, Jansson JK, Knight R. The Earth Microbiome project: successes and aspirations. BMC Biol. 2014;12(69). 10.1186/s12915-014-0069-1 PMC4141107 25184604

[14] Sunagawa S, Coelho LP, Chaffron S, Kultima JR, Labadie K, Salazar G, et al Structure and function of the global ocean microbiome. Science. 2015;348(6237). 10.1126/science.1261359 25999513

[15] Ruff SE, Biddle JF, Teske AP, Knittel K, Boetius A, Ramette A. Global dispersion and local diversification of the methane seep microbiome. Proceedings of the National Academy of Sciences. 2015;112(13):4015–20. 10.1073/pnas.1421865112 PMC4386351 25775520

[16] Orcutt BN, Sylvan JB, Knab NJ, Edwards KJ. Microbial Ecology of the Dark Ocean above, at, and below the Seafloor. Microbiol Mol Biol Rev. 2011;75(2):361–422. 10.1128/MMBR.00039-10 21646433 PMC3122624

[17] Ebbe B, Billett DSM, Brandt A, Ellingsen K, Glover A, Keller S, et al Diversity of Abyssal Marine Life Life in the World's Oceans: Wiley-Blackwell; 2010 p. 139–60.

[18] Bik HM, Sung WAY, De Ley P, Baldwin JG, Sharma J, Rocha-Olivares A, et al Metagenetic community analysis of microbial eukaryotes illuminates biogeographic patterns in deep-sea and shallow water sediments. Mol Ecol. 2012;21(5):1048–59. 10.1111/j.1365-294X.2011.05297.x 21985648 PMC3261328

[19] Green J, Bohannan BJM. Spatial scaling of microbial biodiversity. Trends Ecol Evol. 2006;21(9):501–7. 085WA-0009. 16815589 10.1016/j.tree.2006.06.012

[20] Nemergut DR, Costello EK, Hamady M, Lozupone C, Jiang L, Schmidt SK, et al Global patterns in the biogeography of bacterial taxa. Environ Microbiol. 2011;13(1):135–44. 10.1111/j.1462-2920.2010.02315.x ISI:000285876600012. 21199253 PMC5834236

[21] Pommier T, Canback B, Riemann L, Bostrom KH, Simu K, Lundberg P, et al Global patterns of diversity and community structure in marine bacterioplankton. Mol Ecol. 2007;16(4):867–80.: 10.1111/j.1365-294X.2006.03189.x BIOSIS:PREV200700171424. 17284217

[22] Östman Ö, Drakare S, Kritzberg ES, Langenheder S, Logue JB, Lindström ES. Regional invariance among microbial communities. Ecol Lett. 2010;13(1):118–27. 10.1111/j.1461-0248.2009.01413.x 19968693

[23] Amend AS, Oliver TA, Amaral-Zettler LA, Boetius A, Fuhrman JA, Horner-Devine MC, et al Macroecological patterns of marine bacteria on a global scale. J Biogeogr. 2013;40(4):800–11. 10.1111/jbi.12034

[24] Sul WJ, Oliver TA, Ducklow HW, Amaral-Zettler LA, Sogin ML. Marine bacteria exhibit a bipolar distribution. Proc Natl Acad Sci U S A. 2013;110(6):2342–7. 10.1073/pnas.1212424110 23324742 PMC3568360

[25] Brown JH. On the relationship between abundance and distributions of species. Am Nat. 1984;124(2):255–79. WOS:A1984TG38100008.

[26] Gaston KJ. The Multiple Forms of the Interspecific Abundance-Distribution Relationship. Oikos. 1996;76(2):211–20. 10.2307/3546192

[27] Dang H, Li J, Chen M, Li T, Zeng Z, Yin X. Fine-scale vertical distribution of bacteria in the East Pacific deep-sea sediments determined via 16S rRNA gene T-RFLP and clone library analyses. World J Microb Biot. 2009;25(2):179–88.

[28] Li HR, Yu Y, Luo W, Zeng YX, Chen B. Bacterial diversity in surface sediments from the Pacific Arctic Ocean. Extremophiles. 2009;13(2):233–46. 10.1007/s00792-009-0225-7 ISI:000263784200002. 19153801

[29] Tian F, Yu Y, Chen B, Li HR, Yao YF, Guo XK. Bacterial, archaeal and eukaryotic diversity in Arctic sediment as revealed by 16S rRNA and 18S rRNA gene clone libraries analysis. Polar Biol. 2009;32(1):93–103. 10.1007/s00300-008-0509-x ISI:000262536800013.

[30] Schauer R, Bienhold C, Ramette A, Harder J. Bacterial diversity and biogeography in deep-sea surface sediments of the South Atlantic Ocean. ISME J. 2010;4(2):159–70. 10.1038/ismej.2009.106 WOS:000274800300002. 19829317

[31] Jacob M, Soltwedel T, Boetius A, Ramette A. Biogeography of Deep-Sea Benthic Bacteria at Regional Scale (LTER HAUSGARTEN, Fram Strait, Arctic). PLoS ONE. 2013;8(9):e72779 10.1371/journal.pone.0072779 24023770 PMC3759371

[32] Pop Ristova P, Wenzhofer F, Ramette A, Felden J, Boetius A. Spatial scales of bacterial community diversity at cold seeps (Eastern Mediterranean Sea). ISME J. 2015;9(6):1306–18. 10.1038/ismej.2014.217 25500510 PMC4438319

[33] Costello MJ, Cheung A, De Hauwere N. Surface Area and the Seabed Area, Volume, Depth, Slope, and Topographic Variation for the World’s Seas, Oceans, and Countries. Environ Sci Technol. 2010;44(23):8821–8. 10.1021/es1012752 21033734

[34] Amaral-Zetter LA, Artigas LF, Baross J, Bharathi L, Boetius A, Chandramohan D, et al A global census of marine microbes In: McIntyre, editor. Life in the World's Oceans: Diversity, Distribution and Abundance. Oxford: Blackwell Publishing Ltd; 2010.

[35] Huse SM, Dethlefsen L, Huber JA, Welch DM, Relman DA, Sogin ML. Exploring Microbial Diversity and Taxonomy Using SSU rRNA Hypervariable Tag Sequencing. PLoS Genet. 2008;4(11):e1000255 10.1371/journal.pgen.1000255 WOS:000261481000010. 19023400 PMC2577301

[36] Sogin ML, Morrison HG, Huber JA, Mark Welch D, Huse SM, Neal PR, et al Microbial diversity in the deep sea and the underexplored "rare biosphere". Proc Natl Acad Sci U S A. 2006;103:12115–20. 10.1073/pnas.0605127103 WOS:000239701900053. 16880384 PMC1524930

[37] Schloss PD, Westcott SL, Ryabin T, Hall JR, Hartmann M, Hollister EB, et al Introducing mothur: Open-Source, Platform-Independent, Community-Supported Software for Describing and Comparing Microbial Communities. Appl Environ Microbiol. 2009;75(23):7537–41. 10.1128/aem.01541-09 19801464 PMC2786419

[38] Schloss PD, Gevers D, Westcott SL. Reducing the Effects of PCR Amplification and Sequencing Artifacts on 16S rRNA-Based Studies. PLoS ONE. 2011;6(12):e27310 10.1371/journal.pone.0027310 22194782 PMC3237409

[39] Quince C, Lanzen A, Davenport RJ, Turnbaugh PJ. Removing noise from pyrosequenced amplicons. BMC Bioinformatics. 2011;12:38 10.1186/1471-2105-12-38 21276213 PMC3045300

[40] Pruesse E, Quast C, Knittel K, Fuchs BM, Ludwig W, Peplies J, et al SILVA: a comprehensive online resource for quality checked and aligned ribosomal RNA sequence data compatible with ARB. Nucleic Acids Res. 2007;35(21):7188–96. 10.1093/nar/gkm864 17947321 PMC2175337

[41] Gobet A, Boetius A, Ramette A. Ecological coherence of diversity patterns derived from classical fingerprinting and Next Generation Sequencing techniques. Environ Microbiol. 2013:n/a-n/a. 10.1111/1462-2920.12308 PMC4262003 24147993

[42] Fuhrman JA. Microbial community structure and its functional implications. Nature. 2009;459(7244):193–9. 10.1038/nature08058 WOS:000266036100029. 19444205

[43] Galand PE, Casamayor EO, Kirchman DL, Lovejoy C. Ecology of the rare microbial biosphere of the Arctic Ocean. Proc Natl Acad Sci U S A. 2009;106(52):22427–32. 10.1073/pnas.0908284106 ISI:000273178700070. 20018741 PMC2796907

[44] Gobet A, Böer S, Huse SM, Van Beusekom JEE, Quince C, Sogin ML, et al Diversity and dynamics of rare and of resident bacterial populations in coastal sands. ISME J. 2012;6:542–53. 10.1038/ismej.2011.132 21975598 PMC3280144

[45] Gobet A, Quince C, Ramette A. Multivariate Cutoff Level Analysis (MultiCoLA) of large community data sets. Nucleic Acids Res. 2010;38(15):e155 10.1093/nar/gkq545 WOS:000281345900004. 20547594 PMC2926624

[46] Tamames J, Abellan J, Pignatelli M, Camacho A, Moya A. Environmental distribution of prokaryotic taxa. BMC Microbiol. 2010;10(1):85 10.1186/1471-2180-10-85 20307274 PMC2850351

[47] Grahn N, Olofsson M, Ellnebo-Svedlund K, Monstein H-J, Jonasson J. Identification of mixed bacterial DNA contamination in broad-range PCR amplification of 16S rDNA V1 and V3 variable regions by pyrosequencing of cloned amplicons. FEMS Microbiol Lett. 2003;219(1):87–91. 10.1016/s0378-1097(02)01190-4 12594028

[48] Kulakov LA, McAlister MB, Ogden KL, Larkin MJ, O'Hanlon JF. Analysis of Bacteria Contaminating Ultrapure Water in Industrial Systems. Appl Environ Microbiol. 2002;68(4):1548–55. 10.1128/aem.68.4.1548-1555.2002 11916667 PMC123900

[49] Laurence M, Hatzis C, Brash DE. Common Contaminants in Next-Generation Sequencing That Hinder Discovery of Low-Abundance Microbes. PLoS ONE. 2014;9(5):e97876 10.1371/journal.pone.0097876 24837716 PMC4023998

[50] Salter S, Cox MJ, Turek EM, Calus ST, Cookson WO, Moffatt MF, et al. Reagent contamination can critically impact sequence-based microbiome analyses2014 2014-01-01 00:00:00. 10.1186/s12915-014-0087-z PMC4228153 25387460

[51] Legendre L, Legendre P. Numerical Ecology. Amsterdam: Elsevier Science; 1998 853 p.

[52] Mantel N. Detection of disease clustering and a generalized regression approach. Cancer Res. 1967;27(2P1):209-&. WOS:A19679001100001. 6018555

[53] Longhurst A, Sathyendranath S, Platt T, Caverhill C. An estimate of global primary production in the ocean from satellite radiometer data. J Plankton Res. 1995;17(6):1245–71. WOS:A1995RL65700006.

[54] Seiter K, Hensen C, Schroter E, Zabel M. Organic carbon content in surface sediments—defining regional provinces. Deep-Sea Research Part I-Oceanographic Research Papers. 2004;51(12):2001–26. ISI:000226044500012.

[55] Legendre P. Studying beta diversity: ecological variation partitioning by multiple regression and canonical analysis. Journal of Plant Ecology. 2008;1(1):3–8. 10.1093/jpe/rtm001

[56] Oksanen J, Blanchet GF, Kindt R, Legendre P, O'Hara BR. vegan: Community Ecology Package 2010.

[57] Warnes GR, Bolker B, Bonebakker L, Gentleman R, Huber W, Liaw A, et al. gplots: Various R programming tools for plotting data. 2010.

[58] Magnusson A. gmt: Interface between GMT Map-Making Software and R. 2010.

[59] Pante E, Simon-Bouhet B. marmap: A Package for Importing, Plotting and Analyzing Bathymetric and Topographic Data in R. PLoS ONE. 2013;8(9):e73051 10.1371/journal.pone.0073051 24019892 PMC3760912

[60] van Etten J. Package "gdistance" version 1.1–5 CRAN Repository. 2014.

[61] Roesch LF, Fulthorpe RR, Riva A, Casella G, Hadwin AKM, Kent AD. Pyrosequencing-based assessment of soil pH as a predictor of soil bacterial community structure at the continental scale. Applied & Environmental Microbiology. 2007;75:5111–20. 10.1128/AEM.00335-09 PMC2725504 19502440

[62] Galand PE, Potvin M, Casamayor EO, Lovejoy C. Hydrography shapes bacterial biogeography of the deep Arctic Ocean. ISME J. 2009;4(4):564–76. 10.1038/ismej.2009.134 ISI:000275851100011. 20010630

[63] Heijs SK, Laverman AM, Forney LJ, Hardoim PR, van Elsas JD. Comparison of deep-sea sediment microbial communities in the Eastern Mediterranean. FEMS Microbiol Ecol. 2008;64(3):362–77. 10.1111/j.1574-6941.2008.00463.x 18422633

[64] Walsh EA, Kirkpatrick JB, Rutherford SD, Smith DC, Sogin M, D'Hondt S. Bacterial diversity and community composition from seasurface to subseafloor. ISME J. 2015 10.1038/ismej.2015.175 PMC4796937 26430855

[65] Giovannoni SJ, Stingl U. Molecular diversity and ecology of microbial plankton. Nature. 2005;437(7057):343–8. 10.1038/nature04158 WOS:000231849100040. 16163344

[66] Fry JC, Parkes RJ, Cragg BA, Weightman AJ, Webster G. Prokaryotic biodiversity and activity in the deep subseafloor biosphere. FEMS Microbiol Ecol. 2008;66(2):181–96. 10.1111/j.1574-6941.2008.00566.x WOS:000260051300001. 18752622

[67] Inagaki F, Nunoura T, Nakagawa S, Teske A, Lever M, Lauer A, et al Biogeographical distribution and diversity of microbes in methane hydrate-bearing deep marine sediments, on the Pacific Ocean Margin. Proc Natl Acad Sci U S A. 2006;103(8):2815–20. 10.1073/pnas.0511033103 WOS:000235554900061. 16477011 PMC1413818

[68] Teske AP. Microbial communities of deep marine subsurface sediments: Molecular and cultivation surveys. Geomicrobiol J. 2006;23(6):357–68. 10.1080/01490450600875613 WOS:000241195900003.

[69] Webster G, Parkes RJ, Cragg BA, Newberry CJ, Weightman AJ, Fry JC. Prokaryotic community composition and biogeochemical processes in deep subseafloor sediments from the Peru Margin. FEMS Microbiol Ecol. 2006;58(1):65–85. 10.1111/j.1574-6941.2006.00147.x WOS:000240367500007. 16958909

[70] Kouridaki I, Polymenakou PN, Tselepides A, Mandalakis M, Smith KL Jr. Phylogenetic diversity of sediment bacteria from the deep Northeastern Pacific Ocean: a comparison with the deep Eastern Mediterranean Sea. Int Microbiol. 2010;13:143–50. 20890848 10.2436/20.1501.01.119

[71] Li L, Kato C, Horikoshi K. Bacterial diversity in deep-sea sediments from different depths. Biodiversity & Conservation. 1999;8(5):659–77. 10.1023/a:1008848203739

[72] Dyksma S, Bischof K, Fuchs BM, Hoffmann K, Meier D, Meyerdierks A, et al Ubiquitous Gammaproteobacteria dominate dark carbon fixation in coastal sediments. ISME J. 2015;accepted. 10.1038/ismej.2015.257 PMC4872838 26872043

[73] Giovannoni SJ, Vergin KL. Seasonality in Ocean Microbial Communities. Science. 2012;335(6069):671–6. 10.1126/science.1198078 22323811

[74] Rappé MS, Kemp PF, Giovannoni SJ. Phylogenetic diversity of marine coastal picoplankton 16s rRNA genes cloned from the continental shelf off Cape Hatteras, North Carolina. Limnol Oceanogr. 1997;42(5):811–26.

[75] Barberán A, Bates ST, Casamayor EO, Fierer N. Using network analysis to explore co-occurrence patterns in soil microbial communities. ISME J. 2012;6(2):343–51. 10.1038/ismej.2011.119 21900968 PMC3260507

[76] Meadows A, Meadows PS. Bioturbation in deep sea Pacific sediments. J Geol Soc (Lond). 1994;151(2):361–75. 10.1144/gsjgs.151.2.0361

[77] Smith CR. Factors Controlling Bioturbation in Deep-Sea Sediments and Their Relation to Models of Carbon Diagenesis In: Rowe GT, Pariente V, editors. Deep-Sea Food Chains and the Global Carbon Cycle. NATO ASI Series. 360: Springer Netherlands; 1992 p. 375–93.

[78] Smith CR, Demopoulos AWJ. Ecology of the deep Pacific Ocean floor In: Tyler PA, editor. Ecosystems of the World. 28. Amsterdam: Elsevier; 2003.

[79] Smith CR, Levin LA, Koslow JA, Tyler PA, Glover AG. The near future of the deep seafloor ecosystems In: Polunin N, editor. Aquatic ecosystems: trends and global prospects. Cambridge, UK, New York: Cambridge University Press; 2008 p. 334–49.

[80] Jansson JK, Tas N. The microbial ecology of permafrost. Nat Rev Micro. 2014;12(6):414–25. 10.1038/nrmicro3262 Available: http://www.nature.com/nrmicro/journal/v12/n6/abs/nrmicro3262.html#supplementary-information. 24814065

[81] Boetius A, Anesio AM, Deming JW, Mikucki JA, Rapp JZ. Microbial ecology of the cryosphere: sea ice and glacial habitats. Nat Rev Micro. 2015;13(11):677–90. 10.1038/nrmicro3522 26344407

[82] Zinger L, Boetius A, Ramette A. Bacterial taxa–area and distance–decay relationships in marine environments. Mol Ecol. 2014;23(4):954–64. 10.1111/mec.12640 24460915 PMC4230465

[83] McClain CR, Hardy SM. The dynamics of biogeographic ranges in the deep sea. Proc R Soc B. 2010;277(1700):3533–46. ISI:000283450100001. 10.1098/rspb.2010.1057 20667884 PMC2982252

[84] Pedros-Alio C. Marine microbial diversity: can it be determined? Trends Microbiol. 2006;14(6):257–63. 10.1016/j.tim.2006.04.007 WOS:000238697300005. 16679014

[85] Klappenbach JA, Saxman PR, Cole JR, Schmidt TM. rrndb: the Ribosomal RNA Operon Copy Number Database. Nucleic Acids Res. 2001;29(1):181–4. PMC29826. 11125085 10.1093/nar/29.1.181 PMC29826

[86] Gaston KJ, Blackburn TM, Lawton JH. Interspecific Abundance-Range Size Relationships: An Appraisal of Mechanisms. J Anim Ecol. 1997;66(4):579–601. 10.2307/5951

[87] Barberán A, Ramirez KS, Leff JW, Bradford MA, Wall DH, Fierer N. Why are some microbes more ubiquitous than others? Predicting the habitat breadth of soil bacteria. Ecol Lett. 2014;17(7):794–802. 10.1111/ele.12282 24751288

[88] Cho JC, Tiedje JM. Biogeography and degree of endemicity of fluorescent Pseudomonas strains in soil. Appl Environ Microbiol. 2000;66(12):5448–56. WOS:000167112400052. 11097926 10.1128/aem.66.12.5448-5456.2000 PMC92480

[89] Fulthorpe RR, Roesch LFW, Riva A, Triplett EW. Distantly sampled soils carry few species in common. ISME J. 2008;2(9):901–10. ISI:000259298200001. 10.1038/ismej.2008.55 18528413

[90] Ranjard L, Dequiedt S, Chemidlin Prévost-Bouré N, Thioulouse J, Saby NPA, Lelievre M, et al Turnover of soil bacterial diversity driven by wide-scale environmental heterogeneity. Nat Commun. 2013;4:1434 10.1038/ncomms2431 23385579

[91] Bell T. Experimental tests of the bacterial distance-decay relationship. ISME J. 2010;4(11):1357–65. 10.1038/ismej.2010.77 20535220

[92] Martiny JBH, Eisen JA, Penn K, Allison SD, Horner-Devine MC. Drivers of bacterial β-diversity depend on spatial scale. Proc Natl Acad Sci U S A. 2011;108(19):7850–4. 10.1073/pnas.1016308108 21518859 PMC3093525

[93] Ghiglione J-F, Galand PE, Pommier T, Pedrós-Alió C, Maas EW, Bakker K, et al Pole-to-pole biogeography of surface and deep marine bacterial communities. Proc Natl Acad Sci U S A. 2012;109(43):17633–8. 10.1073/pnas.1208160109 23045668 PMC3491513

[94] Muller AL, de Rezende JR, Hubert CRJ, Kjeldsen KU, Lagkouvardos I, Berry D, et al Endospores of thermophilic bacteria as tracers of microbial dispersal by ocean currents. ISME J. 2014;8(6):1153–65. 10.1038/ismej.2013.225 24351936 PMC4030223

[95] Hamdan LJ, Coffin RB, Sikaroodi M, Greinert J, Treude T, Gillevet PM. Ocean currents shape the microbiome of Arctic marine sediments. ISME J. 2013;7(4):685–96. http://www.nature.com/ismej/journal/v7/n4/suppinfo/ismej2012143s1.html. 10.1038/ismej.2012.143 23190727 PMC3603395

[96] Hilário A, Metaxas A, Gaudron S, Howell K, Mercier A, Mestre N, et al Estimating dispersal distance in the deep sea: challenges and applications to marine reserves. Frontiers in Marine Science. 2015;2 10.3389/fmars.2015.00006

[97] Soininen J, McDonald R, Hillebrand H. The distance decay of similarity in ecological communities. Ecography. 2007;30(1):3–12. 10.1111/j.0906-7590.2007.04817.x

[98] Bienhold C, Boetius A, Ramette A. The energy-diversity relationship of complex bacterial communities in Arctic deep-sea sediments. ISME J. 2012;6:724–32. 10.1038/ismej.2011.140 22071347 PMC3309351

[99] Fuhrman JA, Steele JA, Hewson I, Schwalbach MS, Brown MV, Green JL, et al A latitudinal diversity gradient in planktonic marine bacteria. Proc Natl Acad Sci U S A. 2008;105(22):7774–8. 10.1073/pnas.0803070105 WOS:000256648600028. 18509059 PMC2409396

[100] Hillebrand H. On the generality of the latitudinal diversity gradient. Am Nat. 2004;163(2):192–211. WOS:000220378700003. 14970922 10.1086/381004

[101] Soininen J, Lennon JJ, Hillebrand H. A multivariate analysis of beta diversity across organisms and environments. Ecology. 2007;88:2830–8. WOS:000251067900020. 18051652 10.1890/06-1730.1

[102] Ladau J, Sharpton TJ, Finucane MM, Jospin G, Kembel SW, O'Dwyer J, et al Global marine bacterial diversity peaks at high latitudes in winter. ISME J. 2013;7(9):1669–77. 10.1038/ismej.2013.37 23514781 PMC3749493

[103] Boetius A, Damm E. Benthic oxygen uptake, hydrolytic potentials and microbial biomass at the Arctic continental slope. Deep-Sea Res, Part I. 1998;45(2–3):239–75. ISI:000074794300003.

[104] Levin LA, Etter RJ, Rex MA, Gooday AJ, Smith CR, Pineda J, et al Environmental influences on regional deep-sea species diversity. Annu Rev Ecol Syst. 2001;32:51–93. WOS:000172908800003.

[105] Smith CR, De Leo FC, Bernardino AF, Sweetman AK, Arbizu PM. Abyssal food limitation, ecosystem structure and climate change. Trends Ecol Evol. 2008;23(9):518–28. WOS:000259283600011. 10.1016/j.tree.2008.05.002 18584909

