# Supplementary material for: Diversity and Biogeography of Bathyal and Abyssal Seafloor Bacteria
Source: PLoS One. 2016 Jan 27;11(1):e0148016. doi: 10.1371/journal.pone.0148016 (PMC4731391; doi:10.1371/journal.pone.0148016)
Supplement: S1 Fig — Standard deviations for richness are indicated in black. Water depth of each sample is displayed in red (right y axis). No significant relationship was found between richness and water depth (Spearman’s ρ = -0.33 and -0.37 for a and b, respectively, P > 0.05 in both cases). (PDF) [file pone.0148016.s001.pdf]

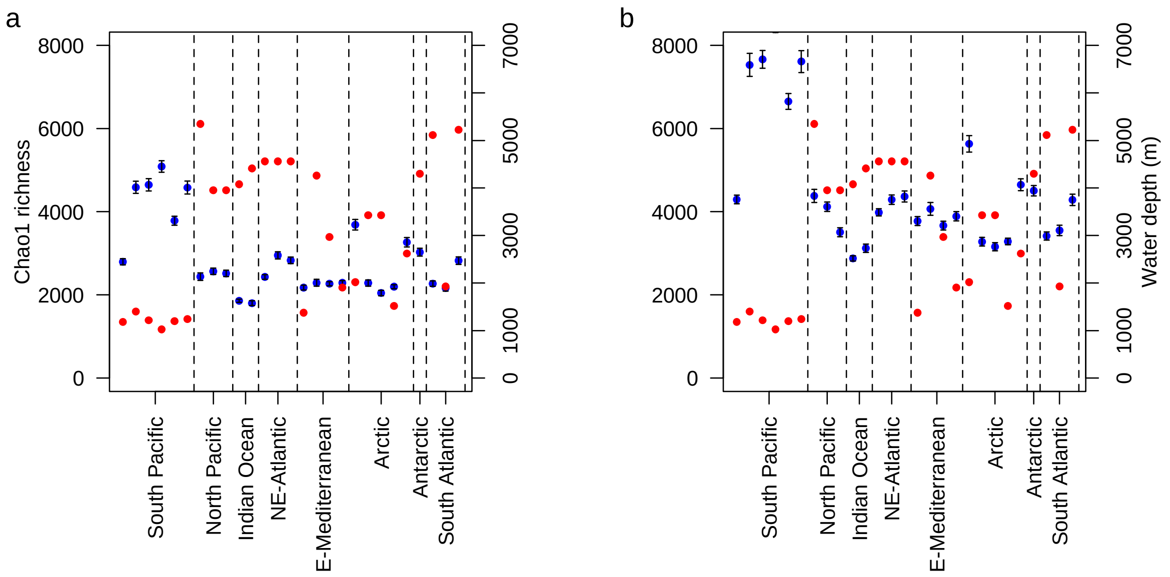

S1 Fig. Chao1 richness estimates (blue, left y axis) calculated with 100 sequence re-samplings for data without (a) and with (b) SSOabs (n resampling = 6,883 and 7,922 sequences for a and b, respectively). Standard deviations for richness are indicated in black. Water depth of each sample is displayed in red (right y axis). No significant relationship was found between richness and water depth (Spearman's ρ = -0.33 and -0.37 for a and b, respectively, $P > 0.05$ in both cases).
