# Supplementary material for: Diversity and Biogeography of Bathyal and Abyssal Seafloor Bacteria
Source: PLoS One. 2016 Jan 27;11(1):e0148016. doi: 10.1371/journal.pone.0148016 (PMC4731391; doi:10.1371/journal.pone.0148016)
Supplement: S3 Fig — Colors in a) mark the taxonomic categories phylum: white, class: red, order: orange, family: yellow. The boxplots show a summary of 100 permutations, calculated with random subsampling, including absolute singletons for comparison. (PDF) [file pone.0148016.s003.pdf]

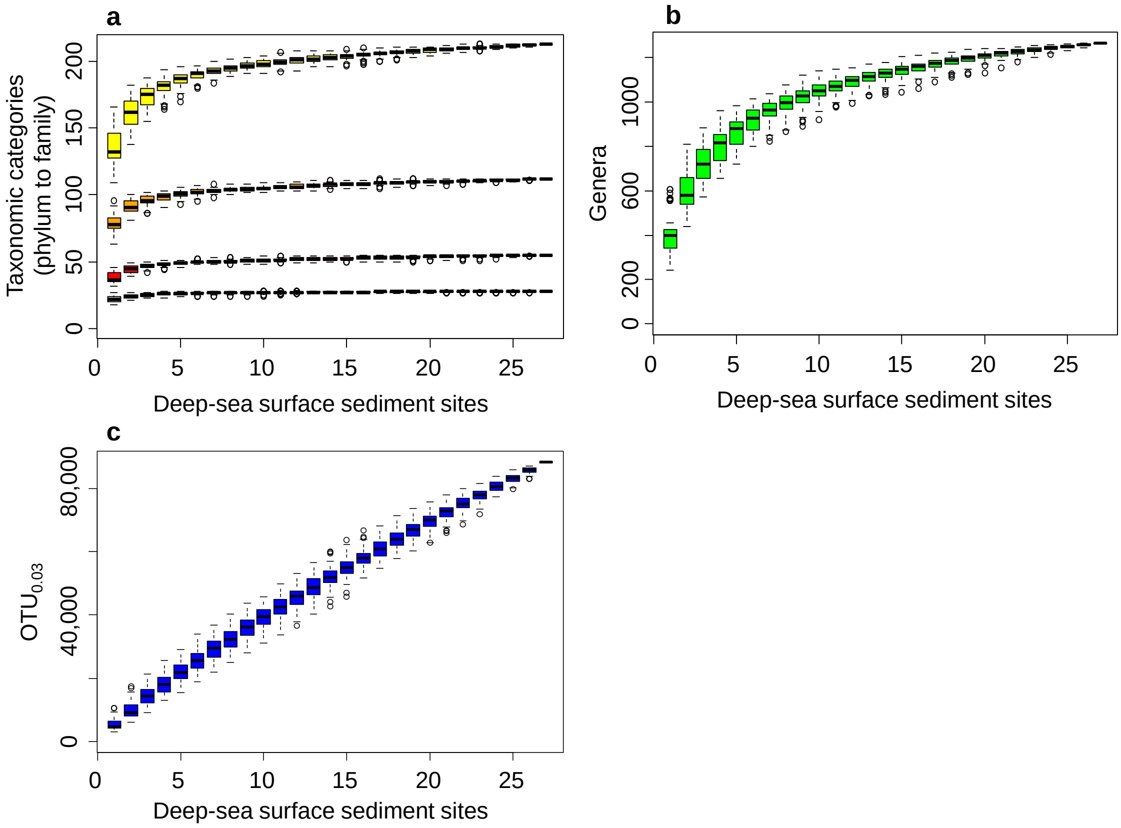

S3 Fig. Species accumulation curves based on different bacterial taxonomic categories: phylum to family (**a**), genera (**b**), and $OTU_{0.03}$ level (**c**). Colors in **a**) mark the taxonomic categories phylum: white, class: red, order: orange, family: yellow. The boxplots show a summary of 100 permutations, calculated with random subsampling, including absolute singletons for comparison.
