# Supplementary material for: Diversity and Biogeography of Bathyal and Abyssal Seafloor Bacteria
Source: PLoS One. 2016 Jan 27;11(1):e0148016. doi: 10.1371/journal.pone.0148016 (PMC4731391; doi:10.1371/journal.pone.0148016)
Supplement: S4 Fig — Relative abundances were averaged across samples and oceans. Error bars indicate standard deviations when considering samples from one oceanic region. (PDF) [file pone.0148016.s004.pdf]

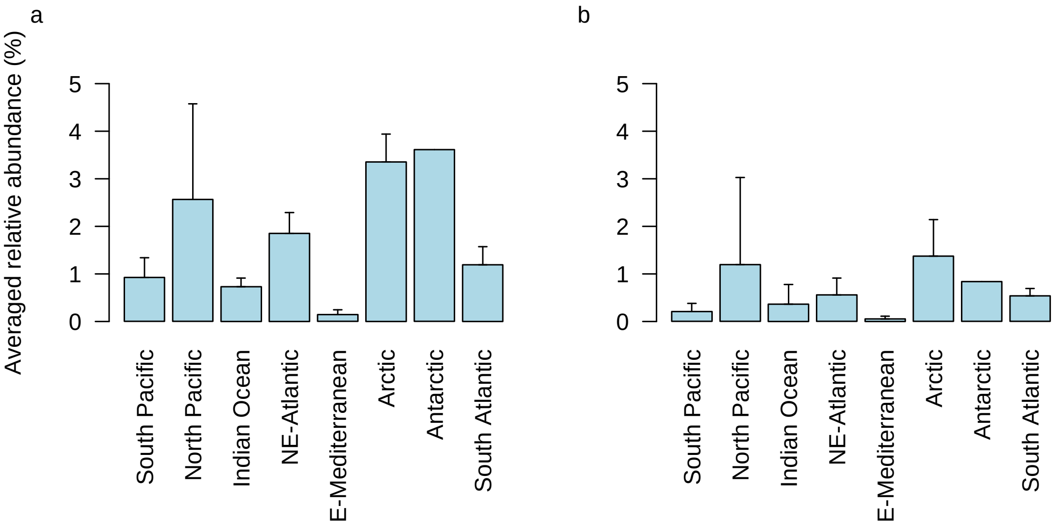

S4 Fig. Variations of truly cosmopolitan $OTU_{0.03}$ affiliated with the clades JTB255 (a, class *Gammaproteobacteria*, n=2) and OM1 (b, class *Actinobacteria*, n=1) between oceanic regions. Relative abundances were averaged across samples and oceans. Error bars indicate standard deviations when considering samples from one oceanic region.
