# Supplementary material for: Diversity and Biogeography of Bathyal and Abyssal Seafloor Bacteria
Source: PLoS One. 2016 Jan 27;11(1):e0148016. doi: 10.1371/journal.pone.0148016 (PMC4731391; doi:10.1371/journal.pone.0148016)
Supplement: S5 Fig — Non-metric multidimensional scaling plots for community composition at the class (a-b) and OTU0.03 (c-d) levels in terms of presence/absence (a,c; using the Jaccard index) and relative abundance (b,d; using the Bray-Curtis index). Samples originating from a same oceanic region are connected by a coloured line, as follows: black: South Pacific; red: North Pacific (St. M); green: Indian Ocean; blue: NE-Atlantic; light-blue: E-Mediterranean; pink: Arctic; yellow: North Pacific (Japan); brown: Antarctic; orange: South Atlantic. Differences in community composition were weak at the class level (ANOSIM R = 0.2 and 0.51, p = 0.03 and 0.001 for a and b, respectively), but significant at the OTU0.03 level (ANOSIM R = 0.7 and 0.66, p = 0.001 for c and d, respectively). (PDF) [file pone.0148016.s005.pdf]

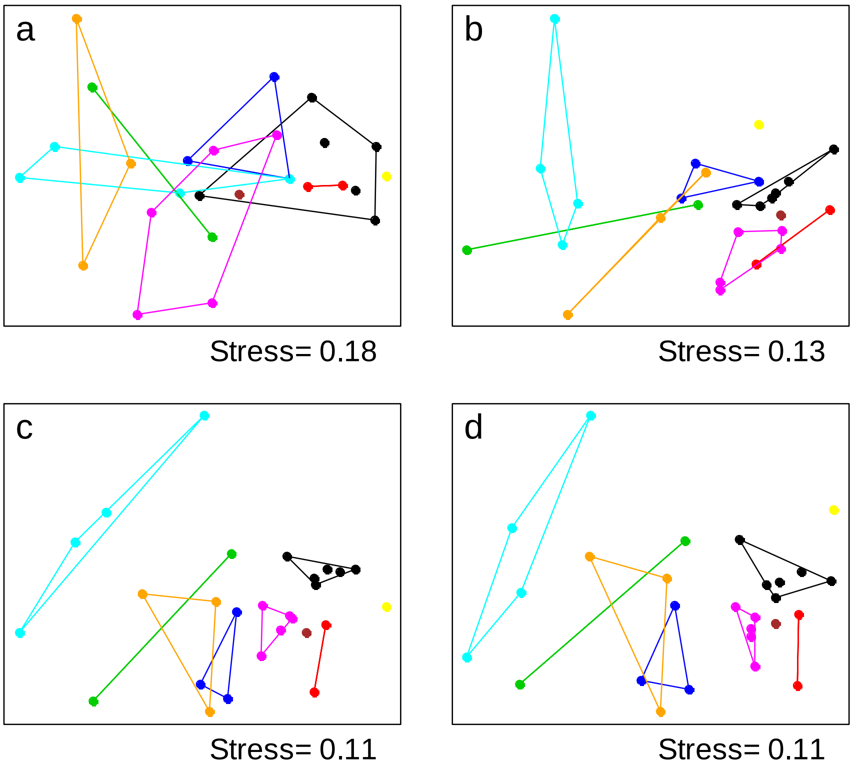

S5 Fig. Differences in bacterial community composition between oceanic regions. Non-metric multidimensional scaling plots for community composition at the class (**a-b**) and $OTU_{0.03}$ (**c-d**) levels in terms of presence/absence (a,c; using the Jaccard index) and relative abundance (b,d; using the Bray-Curtis index). Samples originating from the same oceanic region are connected by a coloured line as follows: black: South Pacific; red: North Pacific (St. M); green: Indian Ocean; blue: NE-Atlantic; light-blue: E-Mediterranean; pink: Arctic; yellow: North Pacific (Japan); brown: Antarctic; orange: South Atlantic. Differences in community composition were weak at the class level (ANOSIM R=0.2 and 0.51, p=0.03 and 0.001 for a and b, respectively), but significant at the $OTU_{0.03}$ level (ANOSIM R=0.7 and 0.66, p=0.001 for c and d, respectively).
