# Supplementary figures and images for: Diversity and Biogeography of Bathyal and Abyssal Seafloor Bacteria
Source: PLoS One. 2016 Jan 27;11(1):e0148016. doi: 10.1371/journal.pone.0148016 (PMC4731391; doi:10.1371/journal.pone.0148016)

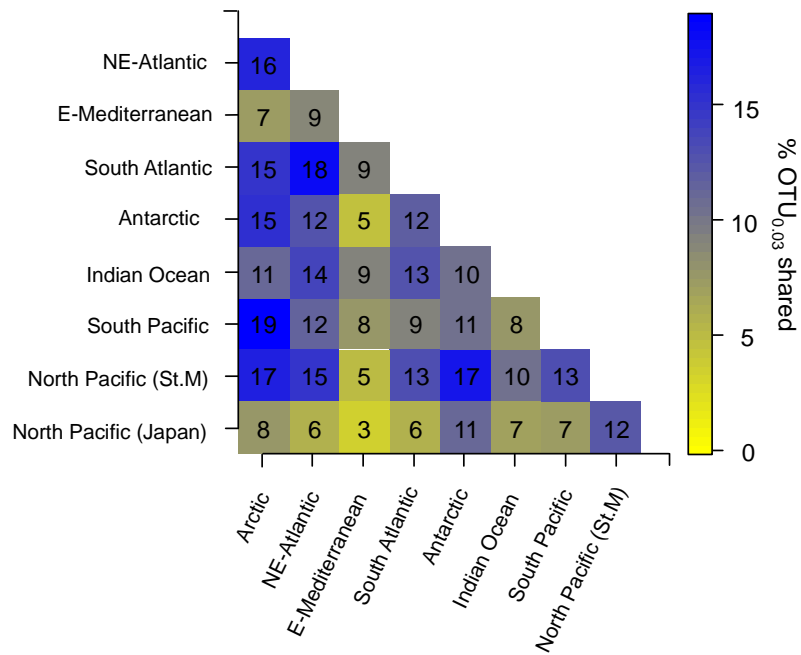

**S6 Fig.** Percentage of shared OTU<sub>0.03</sub> between oceanic regions.

Supplement: S6 Fig — (PDF) [file pone.0148016.s006.pdf]
