# Supplementary material for: Diversity and Biogeography of Bathyal and Abyssal Seafloor Bacteria
Source: PLoS One. 2016 Jan 27;11(1):e0148016. doi: 10.1371/journal.pone.0148016 (PMC4731391; doi:10.1371/journal.pone.0148016)
Supplement: S7 Fig — (PDF) [file pone.0148016.s007.pdf]

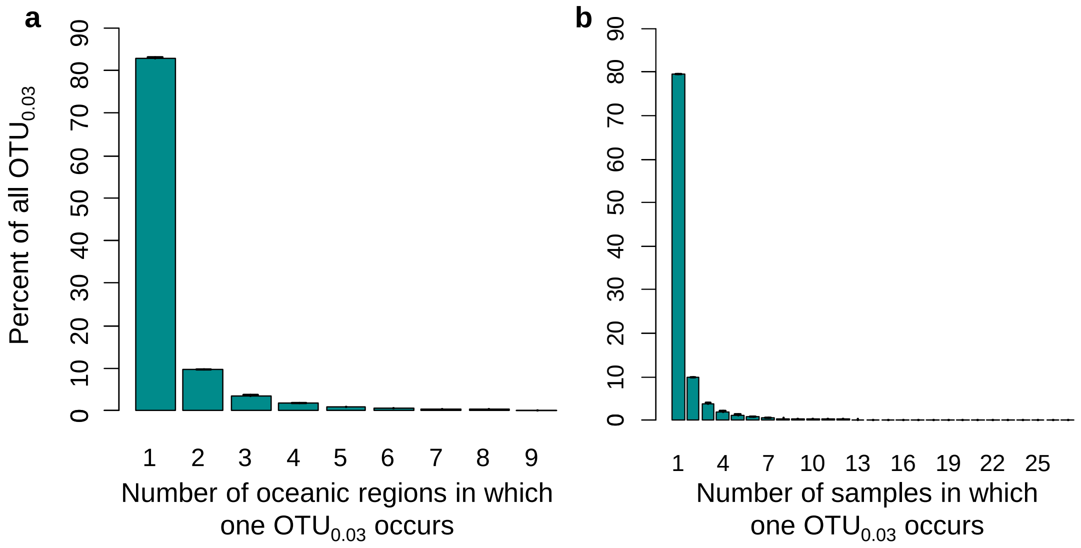

S7 Fig. Proportions of unique and cosmopolitan $OTU_{0.03}$ between oceanic regions and individual samples, including SSOabs, and after averaging of 100 sequence random resampling results (n sequences = 7,922, standard deviations are indicated).
