# Supplementary material for: Diversity and Biogeography of Bathyal and Abyssal Seafloor Bacteria
Source: PLoS One. 2016 Jan 27;11(1):e0148016. doi: 10.1371/journal.pone.0148016 (PMC4731391; doi:10.1371/journal.pone.0148016)
Supplement: S8 Fig — ** p = 0.01, * p = 0.05, (*) p = 0.07, as tested with 100 permutations. (PDF) [file pone.0148016.s008.pdf]

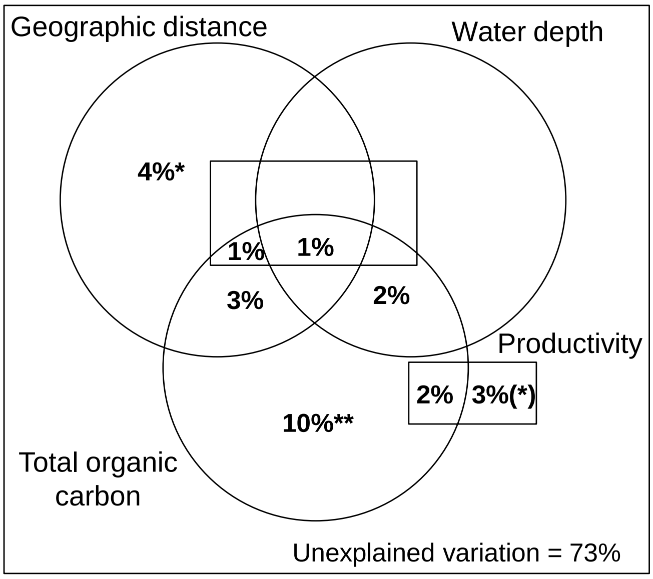

Full model p = 0.001 ***

S8 Fig. Partitioning of the biological variation in bacterial community structure at the $OTU_{0.03}$ level (with absolute singletons excluded) between the following contextual parameters: geographic distance between samples, water depth, TOC availability (TOC regions based on Seiter et al. 2004, Deep Sea Res., Part I **51:** 2001-2026), and surface productivity (Longhurst productivity index based on Longhurst et al. 1995, J Plankton Res **17:** 1245-1271). ** p = 0.01, * p = 0.05, (*) p = 0.07, as tested with 100 permutations.
