# Supplementary material for: Diversity and Biogeography of Bathyal and Abyssal Seafloor Bacteria
Source: PLoS One. 2016 Jan 27;11(1):e0148016. doi: 10.1371/journal.pone.0148016 (PMC4731391; doi:10.1371/journal.pone.0148016)
Supplement: S9 Fig — Linear model is not significant. (PDF) [file pone.0148016.s009.pdf]

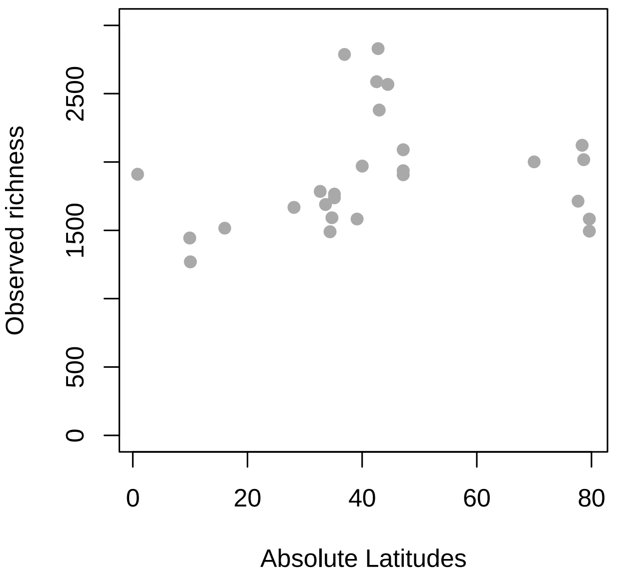

S9 Fig. Bacterial $OTU_{0.03}$ richness (excluding absolute singletons) as a function of latitude. Linear model is not significant.
