# Supplementary material for: Diversity and Biogeography of Bathyal and Abyssal Seafloor Bacteria
Source: PLoS One. 2016 Jan 27;11(1):e0148016. doi: 10.1371/journal.pone.0148016 (PMC4731391; doi:10.1371/journal.pone.0148016)
Supplement: S1 Table — (PDF) [file pone.0148016.s010.pdf]

S1 Table. Contextual data for all deep-sea samples: VAMPS (http://vamps.mbl.edu) sample ID, geographic origin, water depth, oceanic region, and sequence archive accession numbers for GenBank Sequence Read Archives (www.ncbi.nlm.nih.gov).

Sample ID	Longitude	Latitude	Water depth (m)	Date of sampling	Expedition	Oceanic region	Sequence Archive accession #
DSS_0001_1995_10_03	60.268	16.051	4078	10/3/1995	RV Meteor M 33/1	Indian Ocean	SRA046414.1
DSS_0002_1995_10_21	65.034	10.050	4411	10/21/1995	RV Meteor M 33/1	Indian Ocean	SRA046414.1
DSS_0003_1992_03_27	-19.583	47.167	4560	3/27/1992	RV Meteor M 21/1	North-East Atlantic	SRA046414.1
DSS_0004_1992_04_04	-19.583	47.167	4560	4/4/1992	RV Meteor M 21/1	North-East Atlantic	SRA046414.1
DSS_0005_1992_08_05	-19.583	47.167	4560	8/5/1992	RV Meteor M 21/6	North-East Atlantic	SRA046414.1
DSS_0006_1993_05_26	25.859	34.742	1375	5/26/1993	RV Meteor M 25/1	Eastern Mediterranean	SRA046414.1
DSS_0007_1993_05_27	26.097	34.414	4260	5/27/1993	RV Meteor M 25/1	Eastern Mediterranean	SRA046414.1
DSS_0008_1993_05_29	28.571	33.604	2968	5/29/1993	RV Meteor M 25/1	Eastern Mediterranean	SRA046414.1
DSS_0009_1993_05_31	30.597	32.680	1904	6/1/1993	RV Meteor M 25/1	Eastern Mediterranean	SRA046414.1
DSS_0015_1993_09_04	133.191	78.387	2019	9/4/1993	RV Polarstern ARK IX/4	Arctic Ocean (Laptev Sea)	SRA046414.1
DSS_0016_1993_09_03	130.596	79.652	3427	9/3/1993	RV Polarstern ARK IX/4	Arctic Ocean (Laptev Sea)	SRA046414.1
DSS_0017_1993_09_03	130.596	79.652	3427	9/3/1993	RV Polarstern ARK IX/4	Arctic Ocean (Laptev Sea)	SRA046414.1
DSS_0020_1993_09_15	118.577	77.680	1517	9/15/1993	RV Polarstern ARK IX/4	Arctic Ocean (Laptev Sea)	SRA046414.1
DSS_0021_1993_09_14	118.742	78.667	2620	9/14/1993	RV Polarstern ARK IX/4	Arctic Ocean (Laptev Sea)	SRA046414.1
DSS_0022_2006_06_07	143.893	39.106	5347	6/7/2006	RV Yokosuka YK-06-05	North Pacific (Japan)	SRA046414.1
DSS_0023_2007_02_11	177.023	-40.022	1181	2/11/2007	RV Sonne SO 1919	South Pacific (New Zealand)	SRA046414.1
DSS_0033_2005_02_23	-14.000	-70.000	4300	2/23/2005	RV Polarstern ANT XXII/3	Antarctic	SRA046414.1
DSS_0034_2005_03_04	7.347	-28.112	5114	3/4/2005	RV Meteor M 63/2	South-Atlantic (Cape)	SRA046414.1
DSS_0035_2005_03_11	0.897	-9.932	1928	4/11/2005	RV Meteor M 63/2	South-Atlantic (Angola)	SRA046414.1
DSS_0036_2005_03_19	0.833	0.833	5225	4/19/2005	RV Meteor M 63/2	South-Atlantic (Guinea)	SRA046414.1
NZS_0003_2007_04_16	-176.714	-42.782	1025	4/16/2007	https://vamps.mbl.edu/portals/icomm/icomm.php/microbis/	South Pacific (New Zealand)	SRA009906.1
NZS_0004_2007_04_26	175.930	-42.992	1197	4/26/2007	https://vamps.mbl.edu/portals/icomm/icomm.php/microbis/	South Pacific (New Zealand)	SRA009906.1
NZS_0007_2007_04_06	177.141	-44.485	1241	4/6/2007	https://vamps.mbl.edu/portals/icomm/icomm.php/microbis/	South Pacific (New Zealand)	SRA009906.1
NZS_0011_2007_04_20	-178.339	-42.531	1400	4/20/2007	https://vamps.mbl.edu/portals/icomm/icomm.php/microbis/	South Pacific (New Zealand)	SRA009906.1
NZS_0013_2007_05_30	167.526	-36.920	1217	5/30/2007	https://vamps.mbl.edu/portals/icomm/icomm.php/microbis/	South Pacific (New Zealand)	SRA009906.1
SMS_0001_2007_09_19	-123.016	35.164	3953.5	9/19/2007	https://vamps.mbl.edu/portals/icomm/icomm.php/microbis/	North Pacific (Station M)	SRA009865.1
SMS_0004_2007_09_23	-123.016	35.164	3953.5	9/23/2007	https://vamps.mbl.edu/portals/icomm/icomm.php/microbis/	North Pacific (Station M)	SRA009865.1
