# Supplementary material for: Diversity and Biogeography of Bathyal and Abyssal Seafloor Bacteria
Source: PLoS One. 2016 Jan 27;11(1):e0148016. doi: 10.1371/journal.pone.0148016 (PMC4731391; doi:10.1371/journal.pone.0148016)
Supplement: S2 Table — (PDF) [file pone.0148016.s011.pdf]

S2 Table. Genomic bacterial DNA of the 16S rRNA gene V6 hypervariable region was amplified using a cocktail of primers (see also http://vamps.mbl.edu):

Forward primers 967F	Reverse primers 1046R
CTAACCGANGAACCTYACC	CGACAGCCATGCANCACCT
CNACGCGAAGAACCTTANC	CGACGGCCATGCANCACCT
CAACGCGAAAAACCTTACC	CGACGACCATGCANCACCT
CAACGCGCAGAACCTTACC	CGACAACCATGCANCACCT
ATACGCGARGAACCTTACC	
