# Supplementary material for: Diversity and Biogeography of Bathyal and Abyssal Seafloor Bacteria
Source: PLoS One. 2016 Jan 27;11(1):e0148016. doi: 10.1371/journal.pone.0148016 (PMC4731391; doi:10.1371/journal.pone.0148016)
Supplement: S3 Table — a) Twenty most abundant bacterial OTU0.03 for deep-sea surface sediment (> 1000 m water depth). Total number of samples considered is 27. Total number of sequences in the dataset is 501,480. b) Twenty most abundant bacterial OTU0.03 for deep subsurface samples (between 2.5 and 90 m below seafloor from the Peru Margin; http://icomm.mbl.edu). Total number of samples considered is 5. Total number of sequences in the dataset is 72,294. (PDF) [file pone.0148016.s012.pdf]

S3a Table. Twenty most abundant bacterial $OTU_{0.03}$ for deep-sea surface sediment (> 1000 m water depth). Total number of samples considered is 27. Total number of sequences in the dataset is 501,480.

Sediment OTU	Taxonomy	Sequence abundance	Relative sequence abundance	Number (and %) of samples in which present
1	Proteobacteria;Betaproteobacteria;Burkholderiales;Burkholderiaceae;Ralstonia	4597	0.0092	21 (78)
2	Proteobacteria;Gammaproteobacteria;Xanthomonadales;JTB255_marine_benthic_group	4513	0.0090	27 (100)
3	Actinobacteria;Acidimicrobiia;Acidimicrobiales;OM1_clade	4226	0.0084	26 (96)
4	Proteobacteria;Gammaproteobacteria;Xanthomonadales;JTB255_marine_benthic_group	4028	0.0080	24 (89)
5	Proteobacteria;Betaproteobacteria;Neisseriales;Neisseriaceae;Formivibrio	3829	0.0076	21 (78)
6	Proteobacteria;Gammaproteobacteria;Xanthomonadales;JTB255_marine_benthic_group	3611	0.0072	26 (96)
7	Planctomycetes;Planctomycetacia;Planctomycetales;Planctomycetaceae;Pir4_lineage	3190	0.0064	18 (67)
8	Proteobacteria;Gammaproteobacteria;Xanthomonadales;JTB255_marine_benthic_group	3119	0.0062	24 (89)
9	Proteobacteria;Betaproteobacteria;Burkholderiales;Burkholderiaceae;Ralstonia	2963	0.0059	20 (74)
10	Actinobacteria;Acidimicrobiia;Acidimicrobiales;OM1_clade	2891	0.0058	23 (85)
11	Actinobacteria;Acidimicrobiia;Acidimicrobiales;OM1_clade	2399	0.0048	27 (100)
12	Proteobacteria;Gammaproteobacteria;Xanthomonadales;JTB255_marine_benthic_group	2316	0.0046	26 (96)
13	Proteobacteria;Betaproteobacteria;Neisseriales;Neisseriaceae;Formivibrio	2283	0.0046	23 (85)
14	Proteobacteria;Betaproteobacteria;Burkholderiales;Burkholderiaceae;Ralstonia	2209	0.0044	20 (74)
15	Actinobacteria;Coriobacteriia;Coriobacteriales;Coriobacteriaceae	1991	0.0040	12 (44)
16	Proteobacteria;Gammaproteobacteria;Xanthomonadales;JTB255_marine_benthic_group	1963	0.0039	27 (100)
17	Planctomycetes;Planctomycetacia;Planctomycetales;Planctomycetaceae;Pir4_lineage	1905	0.0038	20 (74)
18	Bacteroidetes;Flavobacteriia;Flavobacteriales;Flavobacteriaceae;Aestuariibaculum	1596	0.0032	25 (93)
19	Actinobacteria;Acidimicrobiia;Acidimicrobiales;OM1_clade	1542	0.0031	24 (89)
20	Actinobacteria;Actinobacteria;Propionibacteriales;Propionibacteriaceae;Propionibacterium	1521	0.0030	16 (59)

S3b Table. Twenty most abundant bacterial $OTU_{0.03}$ for deep subsurface samples (between 2.5 and 90 m below seafloor from the Peru Margin; http://icomm.mbl.edu). Total number of samples considered is 5. Total number of sequences in the dataset is 72,294.

Subsurface OTU	Taxonomy	Sequence abundance	Relative sequence abundance	Number (and %) of samples in which present
1	Proteobacteria;Betaproteobacteria;Burkholderiales;Comamonadaceae;Diaphorobacter	1703	0.0236	5 (100)
2	Chloroflexi;vadinBA26	1618	0.0224	5 (100)
3	Proteobacteria;Gammaproteobacteria	1476	0.0204	4 (80)
4	Proteobacteria;Gammaproteobacteria;Enterobacteriales;Enterobacteriaceae;Enteric_Bacteria_cluster;Escherichia	1382	0.0191	4 (80)
5	Proteobacteria	1342	0.0186	1 (20)
6	Chloroflexi;vadinBA26	1324	0.0183	5 (100)
7	Chloroflexi;vadinBA26	1186	0.0164	5 (100)
8	Bacteria;Proteobacteria;Gammaproteobacteria;Pseudomonadales;Moraxellaceae;Acinetobacter	880	0.0122	5 (100)
9	Chloroflexi;vadinBA26	851	0.0118	2 (40)
10	Chloroflexi;vadinBA26	830	0.0115	5 (100)
11	Chloroflexi;vadinBA26	774	0.0107	5 (100)
12	Chloroflexi;vadinBA26	655	0.0091	3 (60)
13	Actinobacteria;Actinobacteria;Actinobacteridae;Actinomycetales;Propionibacterineae;Propionibacteriaceae;Propionibacterium	572	0.0079	4 (80)
14	Candidate_division_OP9	561	0.0078	5 (100)
15	Bacteria;unclassified	534	0.0074	5 (100)
16	Bacteria;unclassified	511	0.0071	2 (40)
17	Firmicutes;Bacilli;Bacillales	507	0.0070	4 (80)
18	Proteobacteria;Deltaproteobacteria;Desulfovibrionales;Desulfohalobiaceae;Desulfonauticus	498	0.0069	4 (80)
19	Bacteria;unclassified	493	0.0068	1 (20)
20	Chloroflexi;vadinBA26	493	0.0068	4 (80)
